# The mitoribosome-specific protein mS38 is preferentially required for synthesis of cytochrome *c* oxidase subunits

**DOI:** 10.1093/nar/gkz266

**Published:** 2019-04-10

**Authors:** Jeffri-Noelle Mays, Yolanda Camacho-Villasana, Rodolfo Garcia-Villegas, Xochitl Perez-Martinez, Antoni Barrientos, Flavia Fontanesi

**Affiliations:** 1 Department of Biochemistry and Molecular Biology, University of Miami Miller School of Medicine, Miami, FL 33136, USA; 2 Departamento de Genetica Molecular, Instituto de Fisiologiía Celular, Universidad Nacional Autonoma de Mexico, Mexico City 04510, Mexico; 3 Department of Neurology, University of Miami Miller School of Medicine, Miami, FL 33136, USA

## Abstract

Message-specific translational regulation mechanisms shape the biogenesis of multimeric oxidative phosphorylation (OXPHOS) enzyme in mitochondria from the yeast *Saccharomyces cerevisiae*. These mechanisms, driven mainly by the action of mRNA-specific translational activators, help to coordinate synthesis of OXPHOS catalytic subunits by the mitoribosomes with both the import of their nucleus-encoded partners and their assembly to form the holocomplexes. However, little is known regarding the role that the mitoribosome itself may play in mRNA-specific translational regulation. Here, we show that the mitoribosome small subunit protein Cox24/mS38, known to be necessary for mitoribosome-specific intersubunit bridge formation and 15S rRNA H44 stabilization, is required for efficient mitoribogenesis. Consequently, mS38 is necessary to sustain the overall mitochondrial protein synthesis rate, despite an adaptive ∼2-fold increase in mitoribosome abundance in *mS38*-deleted cells. Additionally, the absence of mS38 preferentially disturbs translation initiation of *COX1, COX2*, and *COX3* mRNAs, without affecting the levels of mRNA-specific translational activators. We propose that mS38 confers the mitochondrial ribosome an intrinsic capacity of translational regulation, probably acquired during evolution from bacterial ribosomes to facilitate the translation of mitochondrial mRNAs, which lack typical anti-Shine-Dalgarno sequences.

## INTRODUCTION

A major challenge in biology is to understand how protein expression is regulated to attend cellular needs. One of the orchestrating layers of regulation involves the translation machinery itself, where protein synthesis is tightly regulated to prevent wasting cellular resources (1). The mitochondrion, a eukaryotic organelle that possesses its own protein synthesis apparatus, has also evolved several mechanisms of translational control, some of which were identified and best studied in the yeast *Saccharomyces cerevisiae* (2). However, the possible role the mitochondrial ribosome plays in these mechanisms and whether it exerts an additional layer of intrinsic regulation remains largely unexplored.

The yeast mitochondrial genome (mtDNA) encodes eight proteins, two ribosomal RNAs (rRNAs) and a set of tRNAs. Seven hydrophobic proteins are components of the multimeric oxidative phosphorylation (OXPHOS) system enzymes, and the eighth is a soluble mitoribosomal protein of the small subunit (mtSSU). Regarding the OXPHOS system, one subunit (Cyt*b*) from the *bc1*-complex or complex III (CIII), three (Cox1, 2 and 3) from the cytochrome *c* oxidase (COX) or complex IV (CIV), and three (Atp6, 8 and 9) from the ATP synthase are encoded in the mtDNA. The remaining subunits of the OXPHOS system complexes, the mitoribosome proteins, and the factors required to assemble these macrostructures are all encoded by the nuclear genome, synthesized in cytoplasmic ribosomes and imported into mitochondria. Therefore, efficient protein synthesis and complex assembly within this organelle require careful coordination to synchronize mitochondrial and cytosolic translation programs (3).

Translation of mitochondrial mRNAs occurs in mitoribosomes, whose composition and structure are adapted to translate mitochondrial mRNAs that lack a typical anti-Shine-Dalgarno element, and to synthesize highly hydrophobic membrane proteins (4). The understanding of these adaptations and their evolution from yeast to humans was recently illuminated by high-resolution cryo-EM reconstructions of the yeast, porcine and human mitoribosomes (5–8). These structures confirmed that some proteins present in bacterial ribosomes have been lost, proteins with homologs in bacteria have substantial extensions, and the mitoribosome has acquired many additional proteins. The assembly lines of the yeast (9) and human mitoribosomes (10) are also starting to emerge and provide insight into the essentiality of mitoribosome proteins for mitoribosome assembly and function.

The yeast mitoribosome small subunit (mtSSU) contains an almost complete set of proteins with homologs in bacteria, with the single exception of bS20 (8). It additionally contains 14 mitochondria-specific proteins, 7 of which have homologs in the mammalian mitoribosome (8). A protein in the latter group is Cox24, the yeast homolog of MRPS38, recently renamed as mS38 (8). Yeast Cox24 was reported to participate in *COX1* mRNA splicing and also translation, as *cox24*-deleted strains carrying intronless mtDNA have attenuated Cox1 synthesis (11). However, the mechanism/s by which Cox24 could influence or regulate *COX1* mRNA-specific translation remained unexplored. Now that Cox24 has been recognized as the mtSSU mS38 protein, the intrigue regarding how it can modulate mRNA-specific translation has only increased.

In the present study, we have used strains carrying intronless mtDNA to analyze the role of mS38 in mitoribosome assembly and function, and its requirement for mRNA-specific translation. We have demonstrated that mitoribosomes lacking mS38 are capable of synthesizing all proteins, although at a strongly attenuated overall mitochondrial translation rate. Importantly, we show that mS38 is preferentially required for translation initiation of not only *COX1* but also *COX2* and *COX3* mRNAs. The mechanism does not directly involve mRNA-specific translational activators, but it involves functional interactions of mS38 with the 5′UTRs of *COX1, COX2* and *COX3* mRNAs. Mitoribosome loading onto these mRNAs can occur in the absence of mS38, but their translation is inhibited. Our results suggest that mS38 was acquired by the mitoribosome before or concurrently with the remodeling of the mRNA channel to facilitate the translation of mitochondrial transcripts. The influence of mS38 on the flexibility of elements forming the mRNA channel and the interaction of mS38 with the *COX1–2-3* mRNAs would be required for the efficient translation of these mRNAs. In this way, our data support the concept of mitoribosome-mediated translational regulation.

## MATERIALS AND METHODS

### Yeast strains and media

All *S. cerevisiae* strains used are listed in supplemental Table S1. The composition of the standard culture medium used is defined in the Supplemental material.

### Antibodies

Several attempts to obtain antibodies against an mS38 peptide were unsuccessful. However, a working antibody against purified recombinant mS38 was obtained using the services of GenScript. The antibody, however, produced multiple crossreacting bands in immunoblotting assays (Supplementary Figure S3A). Therefore, a GST-tagged version of mS38 was generated. A list of all antibodies used in this study is presented in supplemental Table S2.

### Characterization of the mitochondrial respiratory chain and oxidative phosphorylation system

Endogenous cell respiration was assayed in whole cells in the presence of galactose using a Clark-type polarographic oxygen electrode from Hansatech Instruments (Norfolk, UK) at 30°C as described (12).

Mitochondria were prepared from the different strains as described (13) and used for spectrophotometric assays performed at 24°C to measure KCN-sensitive COX activity and antimycin A-sensitive NADH cytochrome *c* reductase, as described (12).

The abundance of OXPHOS complexes in mitochondrial extracts obtained in the presence of 1% lauryl-maltoside was analyzed by Blue Native polyacrylamide gel electrophoresis (BN-PAGE) using a linear 3–12% acrylamide gradient gel (14).

### *In vivo* mitochondrial protein synthesis

Mitochondrial gene products were labeled with [^35^S]-methionine (7 mCi/mmol, Perkin Elmer) in whole cells at room temperature in the presence of 0.2 mg/ml cycloheximide to inhibit cytoplasmic protein synthesis (12). In most experiments, we used 10 μCi [^35^S]-methionine for a final methionine concentration of 17.2 nM. In some time-course experiments, we used a mixture of labeled (2.5 μCi; 4.3 nM) + cold methionine (12.9 nM). When indicated, mitochondrial translation was inhibited by addition of 0.08 mg/ml puromycin and 40 mM cold methionine and samples were chased for the indicated times. Equivalent amounts of total cellular proteins were separated by SDS-PAGE on a 17.5% polyacrylamide gel, transferred to a nitrocellulose membrane and exposed to X-ray film.

### Sucrose gradients

The sedimentation properties in sucrose gradients of mS38, translational activators and mitoribosomal proteins from total mitochondrial extracts were analyzed essentially as previously described (13,15) and are further described in the supplemental experimental procedures.

### Analysis of mitochondrial polysomes

Yeast cells were grown in medium supplemented with 4 mg/ml chloramphenicol (CAP) for 3 h and in fresh medium for 1 h before proceeding with mitochondrial isolation as described (16–18). Mitochondria were further purified using a step sucrose gradient as described (19). Mitochondrial extracts were prepared in the presence of 0.8% sodium deoxycholate (NaDOC), 10 mM Tris–HCl pH. 7.5, 100 mM NH_4_Cl, 20 mM MgCl_2_ and 200 U of RNaseOUT (Thermo Fisher) and loaded onto 10 ml 10–30% sucrose gradients. The gradients were centrifuged in an SW41 Ti rotor (Beckman) at 32 700 rpm during 2.5 h at 4°C. Subsequently, the gradients were fractionated using a BR-188 Density Gradient Fractionation System (Brandel). Aliquots of each of 28 fractions were used for immunoblotting analysis of mitoribosomal markers. The rest was used for RNA extraction, to analyze the presence of the 15S and 21S rRNAs, and *COX1 and COB* mRNAs by cDNA synthesis followed by RT-PCR amplification.

### RNA analysis

Methods for RNA isolation and quantitative real-time polymerase chain reaction (RT-PCR) are described in the supplemental experimental procedures.

### Statistical analysis

All of the experiments were done at least in triplicate. The data are presented as the means ± S.D. of absolute values or percentages of control. The values for the several parameters studied, obtained for WT and Δ*mS38* mutant strains, were compared by Student's *t*-test. *P* < 0.05 was considered significant.

## RESULTS AND DISCUSSION

### mS38 is a mitoribosome SSU protein specifically required for the efficient synthesis of mtDNA encoded COX subunits

Yeast Cox24 was initially reported to act in *COX1* mRNA splicing and translation (11), and subsequently to be the mitoribosome-specific mtSSU protein mS38 (8). How a mtSSU structural subunit participates in mRNA-specific translation is intriguing. To ascertain the mechanism involved, we utilized for all our experiments a Δ*mS38* strain carrying intronless mtDNA (I^0^), to avoid the described effects on *COX1* mRNA splicing, and started by assessing its ability to support mitochondrial mRNA translation and cellular respiration.

**Figure 1.**
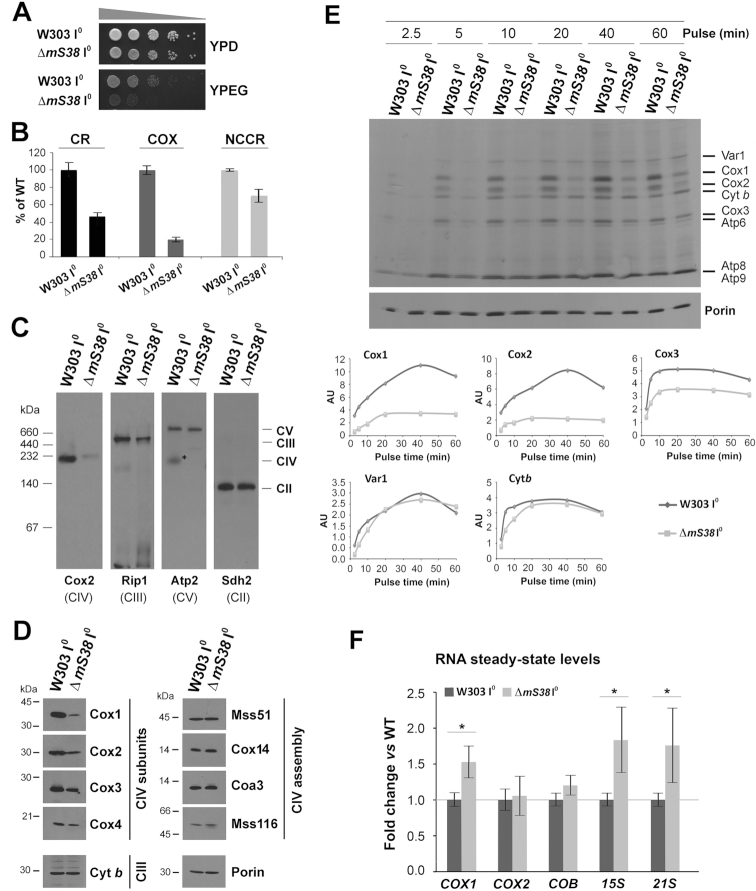
mS38 is specifically required for the efficient translation of *COX1, COX2*, and *COX3* mRNAs. See also Supplementary Figure S1. The intronless WT (W303 I^0^) and Δ*mS38* mutant strains were characterized by: (**A**) Serial dilutions growth test in fermentable complete solid media containing glucose (YPD) and respiratory media containing ethanol and glycerol (YPEG). Pictures were taken after two days of growth at 30°C. (**B**) Measurement of OXPHOS parameters in the indicated cell strains. The graph shows the endogenous cell respiration rate (CR), and the enzymatic activities of CIV or cytochrome *c* oxidase (COX) and of NADH-cytochrome *c* reductase (NCCR), expressed as the percentage of WT. Bars represent the mean ± SD of three independent repetitions. (**C**) Steady‐state levels of OXPHOS complexes extracted from isolated mitochondria with lauryl maltoside, analyzed by BN–PAGE and detected by immunoblotting with the indicated antibodies. (**D**) Immunoblot analyses of the steady-state levels of the indicated complex III (CIII) and complex IV (CIV) subunits and assembly factors. Porin was used as loading control. (**E**) Metabolic labeling with ^35^S-methionine (2.5 μCi; 4.30 nM) + cold methionine (12.90 nM) of newly synthesized mitochondrial products in whole cells during increasing pulse times in the presence of cycloheximide to inhibit cytoplasmic protein synthesis. Immunoblotting for Porin was used as a loading control. Newly-synthesized polypeptides are identified on the right. In the lower panel, raw densitometry values for the indicated individual proteins were plotted to visualize their synthesis kinetics. (**F**) RT-qPCR analysis of mitochondrial RNA steady-state levels in WT (W303 I^0^) and Δ*mS38* cells, expressed as fold change versus WT values. The bars represent the average ± SD of three independent repetitions. Student's *t*-test: *P < 0.01.

As previously reported (11), the Δm*S38* I^0^ strain was found to grow poorly in respiratory YPEG media (Figure 1A). Furthermore, endogenous cell respiration was 45% of WT (Figure 1B). The respiratory defect can be partially attributed to a decrease in the activity of CIII since NADH-cytochrome *c* reductase activity (NCCR) was 70% of WT (Figure 1B). However, the major contributor to the respiratory defect appears to be COX, whose activity was severely decreased to 20% of WT (Figure 1B), in agreement with the markedly low levels of assembled CIV (Figure 1C) and COX subunits (Figure 1D), detected in Δ*mS38* I^0^ mitochondria. The levels of several COX assembly factors were not affected by the *mS38* mutation (Figure 1D). This is particularly relevant for Mss51, a *COX1* mRNA translation activator and Cox1 chaperone (16,20) since the *mS38* gene on chromosome XII of *S. cerevisiae* genome is ‘head to head’ with *MSS51* and, as they share regulatory elements (21), the *mS38* deletion could have affected *MSS51* expression.

To confirm that the OXPHOS biogenesis defect in the Δ*mS38* strain stems from a defect in mtDNA gene expression, we performed *in vivo* mitochondrial translation experiments by following the rate of incorporation of [^35^S]-methionine into the different translation products at increasing pulse times. Our results showed that Δ*mS38* cells could synthesize mitochondrial proteins albeit not efficiently (Figure 1E and Supplementary Figure S1). In the experiment presented in Figure 1E, the labeling reaction was supplemented with cold methionine to slow down and better estimate the rate of [^35^S]-methionine incorporation into newly synthesized polypeptides. As previously reported, the Δ*mS38* mutation most specifically affected synthesis of Cox1 (11), but also of Cox2 and Cox3 (Figure 1E and Supplementary Figure S1). The incorporation of [^35^S]-methionine into Cox1 in the mutant was ∼20% of WT, even at the later time points (Figure 1E and Supplementary Figure S1). For Cox2 and Cox3, labeling was ∼30–70% of WT after 1h of pulse-labeling (Figure 1E). For cytochrome *b* and Var1, although the rate of synthesis in the Δ*mS38* mutant was initially lower than in the WT, in the longer pulses these proteins reached WT labeling saturation (Figure 1E and Supplementary Figure S1).

The poor labeling of COX subunits in the Δ*mS38* mutant strain could be due to an effect of the *mS38* mutation on the rate of protein synthesis or turnover. However, the fact that incorporation of [^35^S]-methionine into Cox1, Cox2 and Cox3 is undetectable after short pulses (Figure 1E, 2.5 min) and that the residual proteins synthesized after 1 h pulse are stable in chase experiments (Supplementary Figure S2A) argues against fast turnover. Likewise, the protein synthesis defect in Δ*mS38* cells is not due to a discrepancy in mRNA levels, as Δ*mS38* I^0^ mitochondria had wild-type levels of *COX2* and *COB* mRNAs, and even enhanced levels of *COX1* mRNA (Figure 1F).

### mS38 is required for translation initiation of *COX1, COX2* and *COX3* mRNAs

The general decrease in mitochondrial protein synthesis rate in the absence of mS38 could be attributed to a change in mtSSU structure near the decoding center and the mRNA exit channel, as well as to the absent intersubunit bridges that involve mS38 (see below). However, translation of a subset of mRNAs, *COX1, COX2* and at a lower extent *COX3*, is deeply affected. These mRNAs might undergo specific interactions with the mS38-containing mtSSU that are missing or attenuated in *ΔmS38* mitochondria.

**Figure 2.**
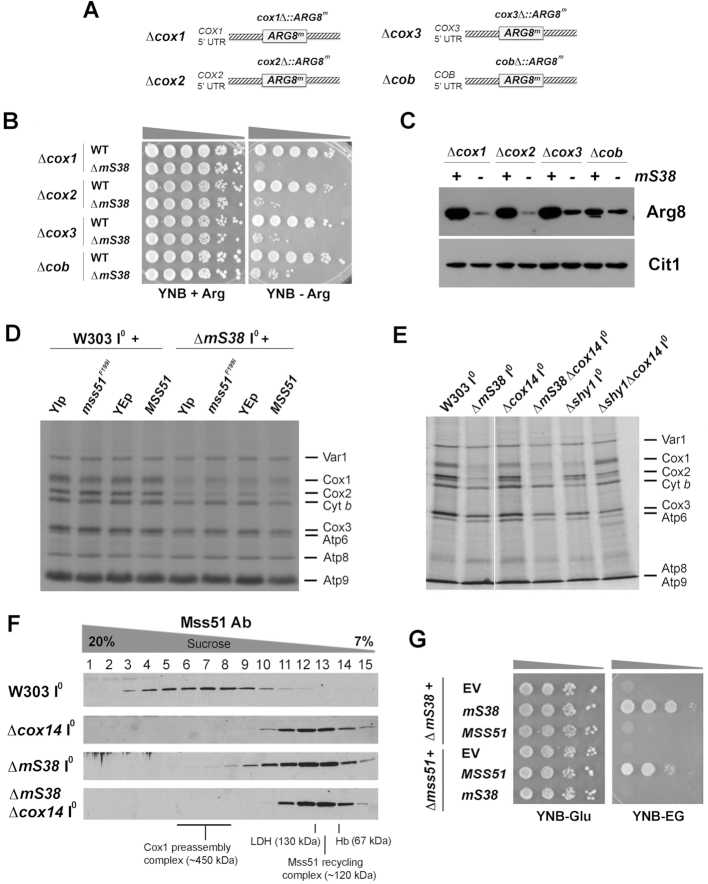
mS38 is required for translation initiation of mtDNA-encoded COX subunits. See also Supplementary Figure S2. (**A**) Scheme depicting the mitochondrial genotype of strains carrying mitochondrial *ARG8* (*ARG8^m^*) as a reporter of mRNA translation. (**B**) Growth test using serial dilutions of the indicated strains in yeast nitrogen base minimum media (YNB) supplemented or not with arginine. Pictures were taken after 2 days of growth at 30°C. (**C**) Steady-state levels of mitochondrial Arg8 in the indicated strains, estimated by immunoblotting. Cit1 (mitochondrial citrate synthase) was used as loading control. (**D** and **E**) *In vivo* mitochondrial protein synthesis performed as in Figure 1E but using ^35^S-labeled (10 μCi; 17.20 nM) and not cold methionine, and the indicated strains. In (**D**), the effect of extra copies of the wild-type *MSS51* or the heme-insensitive allele mss51^F199I^ was tested. YIp: empty integrative vector YIp211. YEp: empty episomal vector YEp351. In (**E**), the effect of a Δ*cox14* mutation was assessed. (**F**) Sucrose gradient sedimentation analyses of translational activator Mss51 on mitochondrial extracts prepared from the indicated strains carrying intronless mtDNA (I^0^) in the presence of 0.8% digitonin. (**G**) Growth test using serial dilutions of the indicated strains in minimum media (YNB) supplemented with fermentable (D, dextrose) or non-fermentable (EG, ethanol-glycerol) carbon sources. Pictures were taken after 2 days of growth at 30°C.

To test the direct involvement of mS38 in the translation of *COX1, COX2* and *COX3* mRNAs, we used strains carrying a mitochondrion-encoded reporter gene (20) in either WT or Δ*mS38*. The strains are deleted for the nuclear *ARG8* gene, and the reporter is a recoded version of *ARG8*, termed *ARG8^m^*, that encodes a matrix-localized biosynthetic enzyme, which allows us to look directly at mitochondrial translation by scoring the growth of yeast in the absence of arginine. In the strain YC162, the *COX1* coding sequence was completely replaced by *ARG8^m^* (*cox1*Δ::*ARG8^m^*). Similarly, *ARG8^m^* gene replaced the *COX2* coding sequence in the strain RGV139 (*cox2*Δ::*ARG8^m^*), the *COX3* coding sequence in the RGV140 strain (*cox3*Δ::*ARG8^m^*), and as a control, the *COB* coding sequence in the RGV137 strain (*cob*Δ::*ARG8^m^*) (Figure 2A). In these strains, *ARG8* mRNA translation is dependent on the 5′- and 3’- untranslated regions (UTRs) of *COX1, COX2, COX3* and *COB* respectively (20). When carrying a WT allele of *mS38*, Arg8^m^ is synthesized in all strains and supports arginine-independent growth (Figure 2B). However, the Δ*mS38* mutation affected capacity of the strains to grow in the absence of exogenous arginine (Figure 2B) due to the general translation defect observed in the Δ*mS38* strain (Figure 1E). The deleterious effect was particularly prominent in the Δ*cox1* and Δ*cox2* strains, less prominent in the Δ*cox3* strain, and markedly less severe in the Δ*cob* control strain (Figure 2B). The growth results correlate well with the steady-state levels of Arg8^m^ detected in each of these strains (Figure 2C). These results indicated that whereas mitochondrial translation in mS38-less mitoribosomes is generally compromised, they are preferentially impaired in translation initiation of *COX1, COX2* and *COX3* RNAs.

### The mS38-dependent Cox1 synthesis defect is not the result of Cox1 translational regulation

Synthesis of Cox1 is coupled to its assembly into COX by a translational negative feedback loop in which Mss51 plays an essential role (16,20). Mss51 acts on the *COX1* mRNA 5′-UTR to promote translation initiation and forms a complex with newly synthesized Cox1 that is stabilized by two specific chaperones, Cox14 and Coa3. Interactions with these chaperones trap Mss51 in a ∼450 kDa Cox1 pre-assembly complex until Cox1 is matured or interacts with its assembly partners (15,20,22–24). When this occurs, Mss51 is released from its interaction with the Cox1 polypeptide, forms a ∼120 kDa recycling complex with the Hsp70 chaperone (Ssc1) and becomes available to promote new rounds of *COX1* mRNA translation (15). To further test whether the decrease in Cox1 synthesis that we observed in the *mS38* mutant is due to a primary defect in translation and not the result of Cox1 synthesis downregulation, we followed two strategies to bypass feedback regulation of *COX1* mRNA translation. First, we previously reported that additional copies of wild-type *MSS51* or a single copy of the mutant allele *mss51*^F199I^ increases Cox1 synthesis in COX assembly mutants in which Cox1 synthesis is downregulated (12,16). As shown in Figure 2D, these strategies did not alleviate the Cox1 synthesis defect observed in the Δ*mS38* strain. Second, we introduced a Δ*cox14* mutation in the Δ*mS38* strain. In most COX assembly mutants, the absence of Cox14 destabilizes the complex of sequestered Mss51 with newly synthesized Cox1 and renders Mss51 available for translation, thus bypassing the regulatory feedback mechanism (16). As shown in Figure 2E, the absence of Cox14 suppressed the Cox1 synthesis downregulation in the control COX mutant strain Δ*shy1* but not in the Δ*mS38* strain. *SHY1* codes for the SURF1 homolog of yeast, a known conserved COX assembly factor (12). The additional Δ*cox14* mutation was not found deleterious for the residual Cox1 synthesis capacity retained by the Δ*mS38* strain (Figure 2E), as previously reported (11). Instead, using pulse-chase assays, we found that as in the Δ*cox14* strain, newly synthesized Cox1 is also very susceptible to degradation in the double mutant strain (Supplementary Figure S2A).

We subsequently followed the sedimentation pattern of Mss51 in sucrose gradients in Δ*cox14*, ΔmS38 and the double Δ*cox14*Δ*mS38 strain*. In WT mitochondria, most Mss51 sediments in the ∼450 kDa Cox1-preassembly complexes, whereas in Δ*cox14* mitochondria it sediments in the 120 kDa Mss51 recycling complex ((15) and Figure 2F). In the absence of mS38, only traces of Mss51 sediment in the ∼450 kDa complex, and the additional absence of Cox14 exerts an epistatic effect (Figure 2F). Furthermore, mS38 and Mss51 do not interact genetically, as overexpression of each protein does not compensate for the absence of the other in terms of protein synthesis (Figure 2D) and respiratory growth (Figure 2G).

We concluded that the Cox1 synthesis defect in the Δ*mS38* strain does not result from negative-feedback translation regulation, but from a *COX1* mRNA translation impairment affecting initiation according to the Arg8^m^ reporter experiments presented earlier, that can be extended to *COX2* and to a lesser extent also to *COX3* mRNAs.

Specific attenuation of the synthesis or assembly of mtDNA-encoded subunits has been reported in strains carrying point mutations in some mtLSU proteins, although the mechanisms involved differ from those underlying the Δ*mS38* phenotype. Temperature-sensitive point mutations in the mtLSU protein bL34 (such as the highly conserved R95 residue to W) result in specific alterations in the synthesis of Cox1 and Cox3, but normal Cox2 synthesis (25). The conserved bL34-R95 residue is in close contact with the 21S rRNA. bL34 also interacts with uL23 and uL24, two components of the polypeptide exit tunnel that was modified from bacteria to become adapted for the passage of hydrophobic polypeptides (5). It has been proposed that the bL34 mutations could slightly modify the exit tunnel cavity and then challenge or block the path for the most hydrophobic polypeptides such as Cox1 and Cox3 (25). Differently, strains expressing variants of mL38, a component of the mtLSU central protuberance, carrying point mutations in its phosphatidylethanolamine-binding protein (PEBP)-like domain *(mL38^Y275^*) undergo normal synthesis of all proteins, even enhanced for Cox1, but have impaired COX assembly (26). The mL38-ts mutation might affect the functional integrity of Coa3, Cox14 and Mss51, three Cox1-binding proteins essential for Cox1 elongation, membrane insertion and stability (26,27).

The phenotype of Δ*mS38* is also reminiscent of that of Δ*mss116*. Mss116 is a DEAD-box helicase that plays distinct roles in *COX1* mRNA splicing, mtLSU biogenesis, and mitochondrial *COX* mRNA-specific translation, predominantly affecting Cox1 synthesis (28). The absence of Mss116 renders unstable the pentatricopeptide repeat protein Pet309, a *COX1* mRNA-specific translational activator, thus explaining the Cox1 synthesis defect. Cox14, Coa3, Mss51 and Mss116 were found stable in Δ*mS38* mitochondria (Figure 1D).

Therefore, mS38 is a mtSSU protein that could directly influence the selection of mitochondrial mRNAs for translation. A potential mechanism could involve mitoribosome heterogeneity, with mitoribosome pools containing or not mS38. However, this possibility is tempered by the fact that over-expression of mS38 in wild-type cells did not affect the synthesis rate of Cox1–3 or any other protein (Supplementary Figure S2B), as it would be expected for a mitoribosomal protein present in all mitoribosomes. Furthermore, the cryo-EM structural reconstruction of the yeast mitoribosome did not display any heterogeneity involving mitoribosomal proteins (8).

### Mitoribosomes are assembled and more abundant in mS38-depleted mitochondria

Recently, mS38 has been identified as a mtSSU subunit by cryo-EM analysis (8). However, the fact that the Δ*mS38* strain can perform mitochondrial translation, despite at a low rate, implies that mitoribosomes are assembled and somehow functional in the absence of mS38.

**Figure 3.**
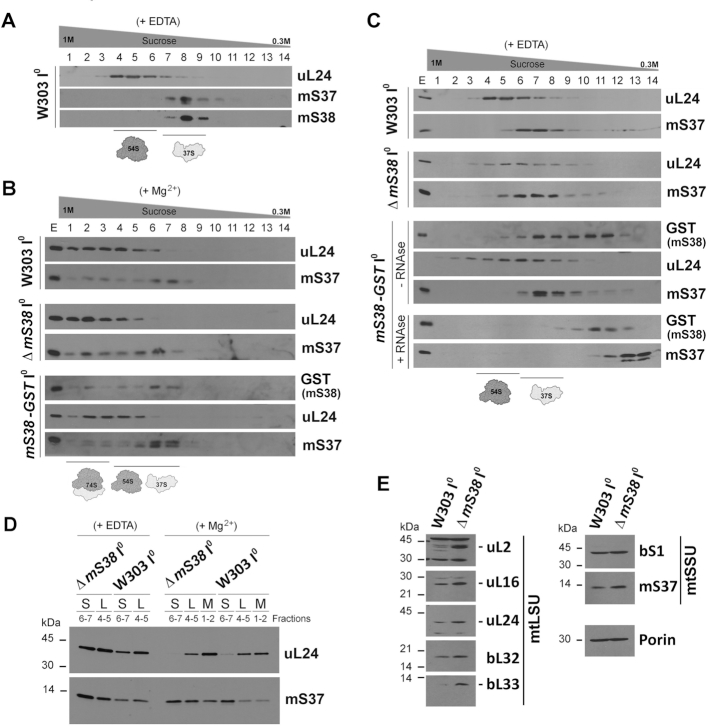
Mitoribosome particles are assembled and more abundant in mS38-depleted mitochondria. See also Supplementary Figure S3. (A–C) Sucrose gradient sedimentation analyses of mitoribosomal protein markers on mitochondrial extracts prepared from the indicated strains carrying intronless mtDNA (I^0^) in the presence of 0.8% digitonin and the conditions stated including either EDTA (**A** and **C**) or Mg^2+^ (**B**). Extracts from a Δ*mS38* strain expressing mS38-GST from an integrative plasmid were prepared in the presence or absence of a high concentration of RNAse (600U) to disrupt mitoribosome integrity. (**D**) To compare the levels of mtSSU (S), mtLSU (L) or monosome (M) in the WT and Δ*mS38* strains, the indicated fractions from the sucrose gradients in panel (B), were pooled and equal volumes analyzed by SDS-PAGE and immunoblotting. (**E**) Steady-state levels of mitoribosomal proteins estimated by immunoblot analyses. Porin was used as a loading control.

To further explore the role mS38 plays in mitoribosome biogenesis and function, we generated a polyclonal antibody against purified recombinant mS38 (Supplementary Figure S3A) that allowed us to determine that in wild-type mitochondria, all endogenous mS38 co-sediments with mtSSU markers (Figure 3A). Similarly, a near fully functional GST-tagged version of mS38 (Supplementary Figure S3B–D) co-sediments with the mtSSU and monosome fractions (Figure 3B) when using extracts prepared in the presence of Mg^2+^. However, when mtSSU and mtLSU are separated by addition of EDTA, a portion of mS38-GST accumulates in a non-assembled pool (Figure 3C), suggesting that under these extraction conditions the large GST tag could destabilize the association of mS38 with the mtSSU. Association of mS38-GST with the mtSSU was further tested by treatment with high concentrations of RNase to disrupt ribosomal integrity. The RNAse treatment resulted in the accumulation of all mS38 in the smaller complex (Figure 3C), further confirming that mS38 is an integral component of the mtSSU.

To probe for a possible mitoribosome assembly defect, mitochondrial proteins were extracted from isolated WT mitochondria using 0.8% digitonin and 25 mM KCl and analyzed by sucrose gradient sedimentation. The sedimentation of the 74S monosome, the 54S mtLSU, and the 37S mtSSU were similar in the WT and Δ*mS38* strains in extracts prepared either in the presence of 0.5 mM MgCl_2_ to preserve the intactness of the monosome or in the presence of 5 mM EDTA to promote subunit dissociation (Figure 3B and C). To obtain a comprehensive picture of potential differences in the composition of assembled mitoribosomal subunits, sucrose gradient fractions from Figure 3C, corresponding to the mtSSU were methanol/chloroform precipitated and analyzed by mass spectrometry (supplemental Table S3). Proteins mS38, mS47 and mS48 were not detected in any of the samples. The rest of the mtSSU proteins were detected in fractions from both WT and Δ*mS38* mitochondrial extracts, which indicates that the absence of mS38 does not prevent incorporation of the remaining proteins into the mtSSU.

To compare the accumulation of mitoribosome protein markers in WT and Δ*mS38* extracts, we ran in parallel, in a single gel, samples from the relevant sucrose gradient fractions (Figure 3D). We determined that the levels of mtSSU and mtLSU proteins were increased in the corresponding fractions and in the fractions where the monosome peaks. In agreement, the steady-state levels of mitoribosome proteins indicated that the concentrations of two mtSSU and five mtLSU proteins tested were enhanced in *ΔmS38* cells compared to the WT control (Figure 3E). Similarly, the steady-state levels of 15S and 21S rRNAs were also found ∼1.5–2-fold increased in Δ*mS38* mitochondria (Figure 1F). We concluded that the steady-state levels of mS38-less mitoribosomes are enhanced, probably to attempt compensating for their inefficient functional performance.

### Mitoribosome loading onto *COX1* mRNA can occur in the absence of mS38

**Figure 4.**
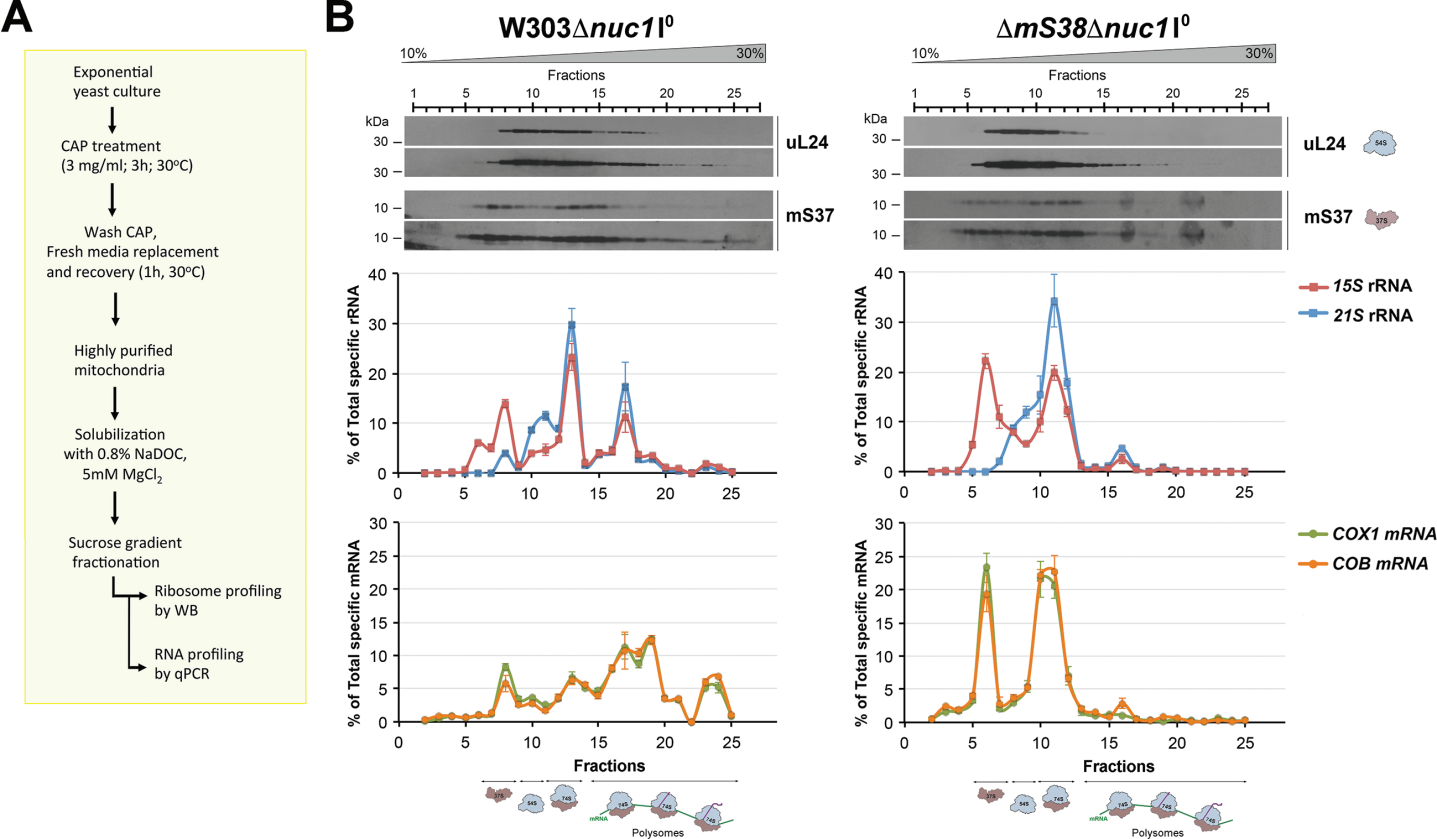
In the absence of mS38, *COX1* mRNA is loaded onto the mitoribosomes but is poorly detected in polysomes. (**A**) Scheme depicting the experimental protocol for mito-polysome profiling. (**B**) Sucrose gradient sedimentation analyses of mitoribosomal subunits, monosomes, and polysomes in extracts obtained from mitochondria isolated from the indicated strains. One representative image of three independent experiments is shown. The graphs in the lower panels represent the relative amount of the indicated rRNAs and mRNAs in the gradient fractions, estimated by RT-qPCR. The bars represent the average ± SD of three independent repetitions.

A failure of translational activators to bind their specific RNA is expected to prevent the proper mRNA loading onto the mitoribosome (29). To assess whether the absence of mS38 limits the mitoribosome-*COX1* mRNA association, we used highly purified mitochondria from WT and Δ*mS38* I^0^ strains, both carrying a null allele of the unspecific mitochondrial nuclease *nuc1*, to avoid RNA degradation during sample processing, as reported (29). Yeast cells were grown in chloramphenicol (CAP) for 3 h and in fresh medium for 1 h prior to mitochondrial isolation (Figure 4A). The CAP treatment allows accumulation of cytoplasmically-synthesized proteins required for mitoribosome assembly and mitochondrial gene expression, which results in a robust increase in the proportion of mito-polysomes and of mitochondrial protein synthesis (16,17,28). Mitochondrial extracts were prepared in the presence of 0.8% NaDOC, 10 mM Tris pH 7.5, 100 mM NH_4_Cl, 20 mM MgCl_2_ and 200 U of RNaseOUT, and fractionated in sucrose gradients. Aliquots of each fraction were used for immunoblot analysis of mitoribosomal markers. The rest was used for RNA extraction and the presence of the 21S and 15S rRNAs, and the *COX1 and COB* mRNAs analyzed by cDNA synthesis followed by quantitative RT-PCR amplification (Figure 4A). Polysomes were detected in the two strains analyzed (Figure 4B). However, they were dramatically decreased in Δ*mS38* mitochondria compared to WT. In our experimental conditions, in both strains, all *COX1* and *COB* mRNAs were found cosedimenting with mitoribosomal particles and not in free pools (Figure 4B). In WT mitochondria, the proportion of *COX1* and *COB* mRNAs detected in the mtSSU was lower than in the monosome and polysome fractions (Figure 4B). On the contrary, in Δ*mS38* mitochondria, the proportion of *COB* and *COX1* mRNAs was higher in the mtSSU and monosome, and attenuated in the polysome fractions, indicating a general role of mS38 in translation. The proportion of *COX1* mRNA on Δ*mS38* polysome fractions was particularly attenuated (Figure 4B), which explains why only traces of newly synthesized Cox1 are detected in mitochondrial protein synthesis experiments (Figure 1E).

Using similar experiments, we previously reported that mitoribosome loading onto *COX1* mRNA could occur in the absence of Pet309 or Mss116 (28). However, the *COX1* mRNA was not detected in polysomes, thus supporting the requirement of Pet309 and Mss116 for *COX1* mRNA translation initiation (28). Similarly, the results presented here indicate the mS38 is not required for the loading of *COX1* mRNA on the mtSSU and the monosome, but rather for its translation.

The mRNA molecules in the cell often assume a secondary and a tertiary structure that might be tight for some genes, and loose for others. For translation to proceed, such structure must first allow ribosome binding and then needs to be threaded through the ribosome, thus providing an opportunity to regulate the translation efficiency of mRNAs (30,31). The 5′-UTRs of *S. cerevisiae* mitochondrial mRNAs are highly variable in length, from 54 nt for *COX2* to 954 nt for *COB*. Their structures have not been determined *in vivo*, where RNA-specific binding proteins are expected to modify them. However, models determined *in silico* by applying free energy rules using the *mfold* web server algorithms have shown that the secondary structures of all naked 5′-UTRs are complex (28). According to these structures, the *COX1* and *COB* 5′-UTRs contain convoluted stem–loops and *COX2* has the shorter and perhaps the simplest 5′-UTR. Although a single short stable hairpin can inhibit ribosome loading at the 5′-end of an mRNA (31), these models suggest that there is more than differences in 5′-UTR arrangements behind the disfavored *COX1–3* mRNAs translation observed in the absence of mS38.

### The preferential inability of mS38-less mitoribosomes to synthesize Cox1 is independent of the *COX1* mRNA-specific translational activators

Next, we investigated whether mS38 genetically or physically interacts with the *COX1* mRNA translational activators, Mss51, Mss116, Pet309, Mam33 and Pet54, which could facilitate the recruitment of the *COX1* mRNA to the mtSSU.

As mentioned above, the five proteins are required for intron splicing on the *COX1* transcript, a function that has also been proposed for mS38 (11), and have been shown to act on the *COX1* mRNA 5′-UTR to promote translation (16,20,28,32). Moreover, Pet54 is a multifunctional protein that acts primarily as a translational activator of *COX3* mRNA and plays a role in Cox1 synthesis possibly by rendering Mss51 competent as a *COX1* mRNA translational activator (33).

**Figure 5.**
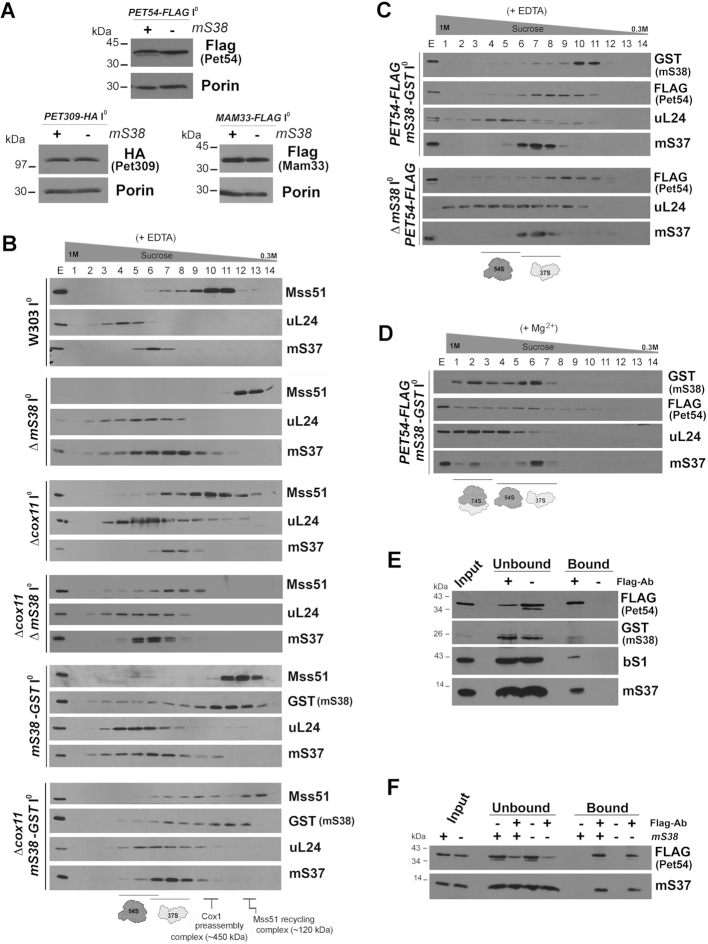
mS38-specific role in regulating *COX* mRNA translation does not directly involve translational activators. See also Supplementary Figure S4 and S5. (**A**) Steady-state levels in wild-type and Δ*ms38* of proteins that regulate *COX1* mRNA translation: Pet54, Pet309 and Mam33. Each strain was constructed by generating double mutants that were reconstituted with an integrated plasmid to express a functional tagged version of the protein being analyzed (Δ*ms38*Δ*pet54 +PET54-FLAG;* Δ*ms38*Δ*pet309 +PET309-HA, and* Δ*ms38*Δ*MAM33 +MAM33-FLAG)*. Porin was used as a loading control. (**B–D**) Sucrose gradient sedimentation analyses of mitoribosomal protein markers and the translational activators Mss51 (B) or Pet54 (C-D) on mitochondrial extracts prepared from the indicated strains carrying intronless mtDNA (I^0^) in the presence of 0.8% digitonin and either EDTA (B, C) or Mg^2+^ (D). (**E**) Co-immunoprecipitation of mS38-GST and Pet54-FLAG, using anti-FLAG-conjugated agarose beads. Protein A-conjugated beads were used as a negative control. (**F**) Co-immunoprecipitation of Pet54-Flag with a mtSSU marker in cells carrying WT or deletion alleles of *mS38* performed as in panel (E).

We started by testing whether overexpression of each translation activator suppresses the Δ*mS38* phenotype. Extra copies of *MSS51* (Figure 2D-G and Supplementary Figure S5C), *MSS116* (data not shown), *MAM33* (Supplementary Figure S5C) or *PET54* (Supplementary Figure S4F-G) failed to restore the respiratory growth or protein synthesis defects observed in the Δ*mS38* strain. Furthermore, the steady-state levels of endogenous Mss51 and Mss116 (Figure 1D) and functional tagged Pet54, Pet309 and Mam33 (Figure 5A, Supplementary Figure S4A and B and Supplementary Figure S5A and B), were not affected by the presence or absence of mS38. Additionally, no additive effect on the rate of Cox1 synthesis was observed in a double null mutant strain *Δpet54ΔmS38* compared to the single mutant *ΔmS38* (Supplementary Figure S4E).

Subsequently, we examined the association of the translational activators with mitoribosomes using sucrose gradient sedimentation analyses. A small fraction of Mss51 was found to co-sediment with the mtSSU in mitochondrial extracts prepared in the presence of EDTA from WT or from Δ*mS38* reconstituted with a GST-tagged version of mS38 (Figure 5B). The absence of mS38 abolished the co-sedimentation of Mss51 with 37S mitoribosomal particles (Figure 5B). This observation could suggest an mS38-mediated interaction of Mss51 with these particles. Alternatively, the Mss51-mitoribosome interaction is most probably mediated through newly synthesized Cox1 and then abolished in Δ*mS38* mitochondria in which Cox1 synthesis is strongly attenuated. To solidify this concept we performed similar experiments using strains carrying a Δ*cox11* mutation, since we have previously shown that the interaction of Mss51 with the mitoribosome is particularly prominent in strains deficient in Cox1 maturation or assembly, such as Δ*cox11*, in which Cox1 synthesis is stalled (15). Consistent with our hypothesis, that Mss51 could interact with the ribosome through newly synthesized Cox1, we observed that Mss51 co-sedimented with the 37S mitoribosome in Δ*cox11*Δ*mS38* mitochondrial extracts (Figure 5B). Also, a functional FLAG-tagged version of Pet54 (Supplementary Figure S4A and B) was found to co-sediment with 37S (Figure 5C and D) and 74S mitoribosomal particles (Figure 5D), although its sedimentation properties were not altered in the absence of mS38 (Figure 5C). Immunoprecipitation studies using a strain expressing *PET54-FLAG* (Figure 5E and F) further confirmed that the interaction of Pet54 with mitoribosomes is independent of mS38. Lastly, no sedimentation with the mitoribosomes was observed for a Flag-tagged functional version of Mam33 (data not shown).

As an additional relevant observation, we noted a different sedimentation pattern in sucrose gradients for Mss51 extracted from strains expressing wild-type mS38 or mS38-GST. The interaction of mS38-GST with the mitoribosome is labile, and a portion is removed from the mitoribosome particle in the presence of EDTA (Figures 3C and 5B), suggesting that the fusion protein may not be fully functional. In fact, although mS38-GST fully supports respiratory growth (Supplementary Figure S3B) and largely restores overall mitochondrial protein synthesis, the synthesis of Cox1 (and at a lesser extent also Cox2 and Cox3) is not fully restored (Supplementary Figure S3D and 7D). For this reason, a portion of Mss51 is trapped in the Mss51 recycling complex (Figure 5B). Importantly, the results obtained with the mS38-GST strain further support the predominant requirement of mS38 for the synthesis of COX subunits.

Overall, from the results presented in this section, we conclude that the preferential inability of mS38-less mitoribosomes to synthesize Cox1 is independent of the *COX1* mRNA-specific translational activators. The case reported here differs from other instances in which interplay between translational activators and mitoribosome subunits, has been reported. Such was the case of mutations in bS6 (former Mrp17) reported to compensate for a C-terminal truncation of Pet122, a *COX3* mRNA-specific translational activator (34).

### The absence of mS38 modifies the decoding center in the mtSSU

**Figure 6.**
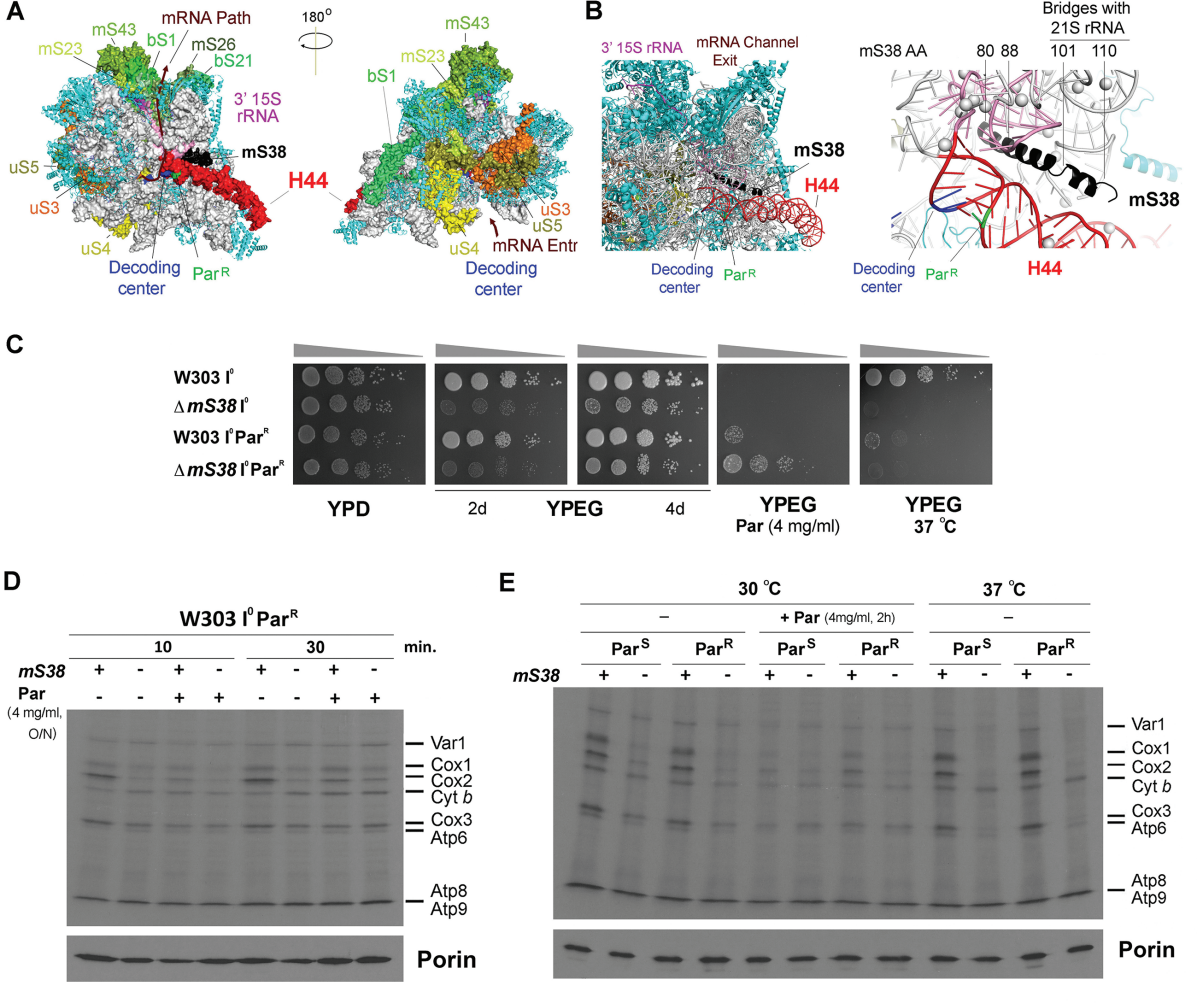
mS38 genetically interacts with mutations in the 15S rRNA helix 44. See also Supplementary Figure S6. (**A**) Localization of the mRNA path in the mtSSU, the proteins located at the entrance and exit channels, as well as mS38, the 15S rRNA helix 44 and the decoding center in the yeast mitoribosome structure (PDB 5MRC) (8). (**B**) Close-up of the mS38-h44 region in the mtSSU structure. The location of the paromomycin-resistance mutation (Par^R^) and key amino acids in mS38 are indicated. (**C**) Serial dilutions growth test of the indicated strains in complete fermentable (YPD) or non-fermentable (YPEG) solid media. The effects of paromomycin supplementation and incubation at 37°C were also tested. Pictures were taken after two days of growth or the time indicated. (D, E) *In vivo* mitochondrial protein synthesis of the indicated strains performed as in Figure 2D. In (**D**), the intronless WT (W303 I^0^) and Δ*mS38* strains with mtDNA carrying the paromomycin-resistant mutation (Par^R^) were incubated or not overnight (ON) in the presence of 4 mg/ml of paromomycin (Par) and pulsed with ^35^S-methionine for 10 or 30 min. In (**E**), the strains with mtDNA carrying paromomycin-sensitive or resistant 15S rRNA sequences were incubated at 30°C in the presence or absence of 4 mg/ml paromomycin for 2 hrs or 37°C and pulsed for 10 minutes with ^35^S-methionine in the same conditions.

In the mtSSU, mS38 interacts with the 15S rRNA helix 44 ((8) Figure 6A-B). This helix is an element present in all ribosomes that forms several inter-subunit bridges, which regulate the relative movement of the subunits, and is part of the decoding center. In bacteria, the lower part of the helix is rigid and anchored by bS20, whereas the middle part forms major intersubunit bridges, and the upper part is flexible being involved in tRNA translocation (35). These H44-involving bridges are also present in the yeast mitoribosome, which contains nine mitochondria-specific bridges, one of which involves mS38 (8). Specifically, mS38 aa residues 101–102, 105–106 and 109–110 connect with 21S rRNA helices H70 (nt 1832–1834), H71 (nt 1859–1860), H62 (nt 1643–1644) and H67 (nt 1739–1741 and 1874–1877), respectively. It has been proposed that the extensive intersubunit bridges may restrain the movement of the yeast mitoribosomal subunits (8). The lack of mS38 in yeast is then predicted to affect subunit association. Additionally, the upper part of H44 is largely more flexible in mitochondrial compared to bacterial ribosomes and its movement is mainly constrained by the presence of mS38. As a consequence, in mS38 absence, H44 is likely to be unrestricted, and decoding could be affected, due to loss of H44 base pairing.

To determine how the absence of mS38 affects H44 near the decoding center we exploited the fact that aminoglycoside antibiotics such as paromomycin bind to the 15S rRNA in the aminoacyl-tRNA site (A-site) to cause misreading of the genetic code and inhibit translocation. Studies in bacteria comparing the 30S subunit with and without paromomycin (36) confirmed an earlier model (37) in which bases 1492 and 1493 of the 16S rRNA are in a position to monitor the codon:anticodon fit via hydrogen bonding to the codon:anticodon duplex. Paromomycin alters the structure of the 16S rRNA to mimic conditions of a perfect codon:anticodon fit, so that incorrect aminoacyl-tRNAs may be selected. In yeast mitochondria, resistance to paromomycin (Par^R^) arises from a single base change in the 15S rRNA H44 region ((39), Figure 6B). This mutation causes a base pair mismatch, which is likely to increase the flexibility of the decoding center. When the Δ*mS38* mutation was introduced into a Par^R^ mtDNA background strain, it did not affect respiratory growth of the resulting strain in regular media (Figure 6C) or its mitochondrial protein synthesis capacity (Figure 6D, E and Supplementary Figure S6) compared to the Par^S^ background. However, the double mutation conferred greater resistance to exogenous paromomycin than the Par^R^ mutation alone (Figure 6C). Notably, the lack of mS38 is associated with strong thermosensitivity. At 37°C, whereas WT mitochondrial translation was unaffected, the synthesis of Cox1, Cox2 and Cox3 was barely detected in Δ*mS38* Par^S^ or Par^R^ backgrounds (Figure 6E), and therefore resistance to exogenous paromomycin was eliminated (Figure 6C). An increase in temperature could also affect the decoding region structure, as reported for *Escherichia coli* ribosomes (38).

Together, these results indicate that the absence of mS38 modifies the decoding center in the mtSSU.

### The mS38 N-terminus is sufficient to support SSU assembly and normal synthesis of all proteins other than COX subunits

**Figure 7.**
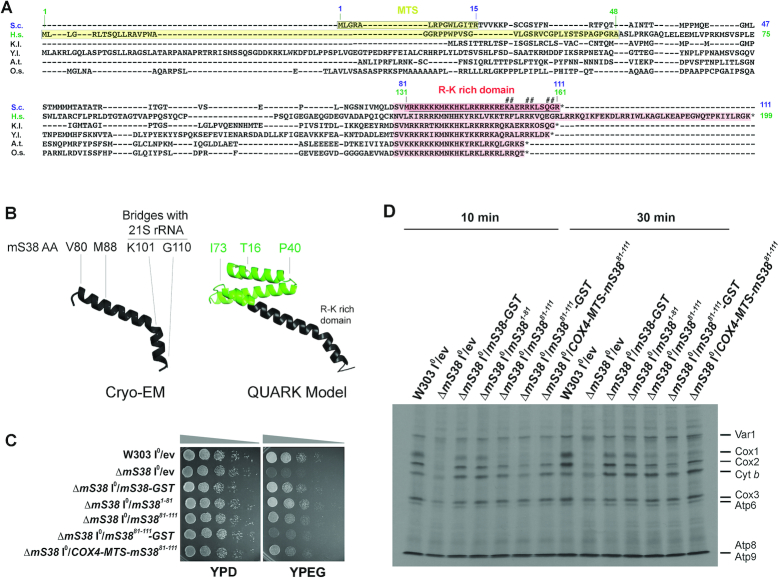
The N- and C-terminal portions of mS38 perform different roles in ribosome assembly and mRNA-specific translation. See also Supplementary Figures S7 and S8. (**A**) Alignment of mS38 sequences from *Saccharomyces cerevisiae (S.c.), Homo sapiens (H.s.)*, other yeasts (*Kluyveromyces lactis*—K.l- and *Yarrowia lipolytica*—Y.l.) and plants (*Arabidopsis thaliana*—A.t., *Oriza sativa*—O.s.). The mitochondrial targeting sequence (MTS) in *S.c* and *H.s*. are marked in yellow. The arginine-lysine (R-G)-rich domain present in the C-terminus of all proteins is labeled in red. Numbers in blue indicate amino acids (aa) in the *S. cerevisiae* protein and numbers in green, amino acids in the human protein. The underlined region in *S. cerevisiae* mS38 is the region that was resolved in the cryo-EM structural reconstruction by (8). The # symbol indicates residues in *S. cerevisiae* mS38 involved in the formation of intersubunit bridge mB3 with elements of the 21S rRNA (8). Bridge mB3 is exclusive of mitochondrial ribosomes but conserved from yeast to mammals. (**B**) Structure of *S. cerevisiae* mS38 as obtained by cryo-EM including amino acids 80–111 (8) and a model of the whole mature protein (aa 16–111) obtained using Quark, a computer algorithm for *ab initio* protein structure prediction (39). Key amino acid residues are indicated. (**C**) Serial dilutions growth test of the indicated strains in complete media containing fermentable (YPD) or non-fermentable (YPEG) carbon sources. Pictures were taken after two days of growth at 30°C. (**D**) *In vivo* mitochondrial protein synthesis using the indicated strains and performed as in Figure 2D following ^35^S-methionine pulses of the indicated increasing times.

*Saccharomyces cerevisiae* mS38 is a 111-aa protein that contains a predicted 15-aa N-terminal mitochondrial targeting sequence (MTS, shadowed in yellow in Figure 7A). The C-terminal aa 81–111 form an arginine-lysine-rich domain (shadowed in red in Figure 7A) conserved from yeast to human. As seen in the cryo-EM structure (8), from the C-terminus, this domain forms a helix that runs parallel to the 15S rRNA H44 at the subunit interface and contains the residues involved in intersubunit bridge formation ((8) and Figure 6B). mS38 then bends, probably restricting the movement of the upper part of H44 that forms the decoding center, and subsequently forces the mS38 N-terminus to protrude into the 15S rRNA (8). The structure of the N-terminal portion of the protein (aa 15–80), which is highly divergent (Figure 7A), was not resolved by cryo-EM. The most probable model of the structure of the full mature protein (aa 16–111), obtained using Quark (Supplementary Figure S8), is shown in Figure 7B. Quark is a computer algorithm for *ab initio* protein structure prediction and protein peptide folding, which aims to construct the correct protein 3D model from amino acid sequence only (39).

Once acquired by the mitoribosome, mS38 probably co-evolved with H44 and the downstream 3’-15S rRNA sequences that mark the mRNA exit path. As an example, the mS38 C-terminus is longer in human than in yeast (Figure 7A). Coevolution was tested by assessing heterologous complementation of the *S. cerevisiae* Δ*mS38* strain with *mS38* from human (also known as AURKAIP1) and several yeast and plant species (Supplementary Figure S7A-C). Human mS38 was unable to substitute for the *S. cerevisiae* protein even when the *S. cerevisiae* mS38 N-terminus was co-expressed (Supplementary Figure S7C). Among fungi, mS38 from *Kluyveromyces lactis* and *Yarrowia lipolytica* complemented the *S. cerevisiae* Δ*mS38* strain. Among plants, mS38 from *Arabidopsis thaliana* did it only partially, and that of *Oriza sativa* did not (Supplementary Figure S7A). These results highlight the structural diversity of mitoribosomes across evolution.

In an attempt to dissect the roles of the mS38 N- and C- termini, each half of the protein was expressed in the Δ*mS38* strain. Expression of either mS38 N-domain (aa 1–81) or mS38 C-domain (aa 1–15+81–111), both containing the 15-aa N-terminal mitochondrial targeting sequence, largely restored the respiratory growth defect of the Δ*mS38* strain (Figure 7C) by repairing its general mitochondrial protein synthesis deficiency (Figure 7D). However, translation of *COX1, COX2* and *COX3* mRNAs remained slightly inefficient in the mS38^1–81^ strain and more attenuated, particularly *COX1* mRNA translation, in the mS38^81–111^ strain (Figure 7D). Similar results were obtained using a strain in which mS38^81–111^ contained the first 15 aa in mS38, predicted to form its mitochondrial targeting sequence (MTS), or when a COX4 MTS was used instead (Figure 7D). In the mS38^1–81^ strain, the stability of newly synthesized polypeptides was as in the wild-type (Supplementary Figure S7D), which suggested that the potential presence of large amounts of aberrant proteins resulting from compromised translational fidelity unable to engage in OXPHOS complex assembly could be disregarded.

Together, the data presented in this section indicates that the N- and C-terminal portions of mS38 are each sufficient to functionally stabilize the mitoribosome and facilitate the translation of most mRNAs, including *COX2* and *COX3*. However, the N-terminal portion of mS38, which according to the structure would be expected to contact mtSSU elements that could influence the transit of mRNA throughout the channel, near the exit site, is particularly important for the translation of *COX1* mRNA.

## CONCLUSION

A requisite property of all ribosomes is their ability to translate genetic information into polypeptides in an accurate and rapid manner. The SSU elements that ensure translation fidelity in bacteria are conserved in mitochondrial and eukaryotic ribosomes, although some adaptations have occurred. In the mitoribosome, these adaptations include the remodeling of the rRNA H44 and surrounding proteins. The mitochondrion-specific SSU protein mS38 was acquired to stabilize the upper part of H44 where the decoding center is located and to establish the critical mitoribosome-specific intersubunit bridge mB4 with the mt-LSU rRNA H71. In this work, we report that *S. cerevisiae* mS38 plays an additional fundamental role in the selection of mRNAs that will be translated.

Our data show that although the absence of mS38 in *S. cerevisiae* attenuates mitochondrial protein synthesis globally, it particularly prevents efficient initiation of *COX1, COX2*, and *COX3* mRNA translation.

The analysis of eukaryotic and bacterial ribosomes across species might provide clues regarding mS38 function. Although mS38 is a mitoribosome-specific protein, it has been noticed by Desai and colleagues (8) that the location and structure of mS38 C-terminus resembles eL41, which is more strongly associated with the eukaryotic SSU 40S subunit than with the LSU 60S subunit and forms a eukaryotic-specific intersubunit bridge (eB14) that stabilizes the monosome (8). Therefore, eL41 and mS38 could be the products of convergent evolution. eL41 is not essential, but its absence reduces the binding efficiency of 60S subunits to translation pre-initiation complexes and also decreases peptidyltransferase activity while enhancing translocation efficiency (40). However, no effect of eL41 on selective mRNA translation has been so far reported.

In bacteria, resolution of the structure of the *Mycobacterium smegmatis* 70S ribosome has revealed the existence of a previously unknown protein, bS22, absent in the *E. coli* ribosome, which folds in a manner and occupies a location beneath the mRNA channel similar to mS38, thus indicating a common evolutionary origin (41). Like mitochondrial mS38, mycobacterial bS22 is rich in arginines and lysines, interacts with the rRNA H44 and probably contributes to its stabilization during translocation of tRNAs. Moreover, it has been proposed that contacts of bS22 with H45 might modify the mRNA channel near its exit site immediately upstream of the anti-Shine-Dalgarno sequence (41). Canonical translation initiation in bacteria requires mRNAs with a 5′-UTR and Shine Dalgarno sequence for ribosome assembly and start codon selection. However, the transcriptome of most bacteria contains 1–2% of leaderless mRNAs, and alternative translation mechanisms have been invoked (42). Remarkably, a quarter of the mycobacterial transcriptome is expressed as leaderless and lack both a 5′-UTR and Shine-Dalgarno sequence (43), raising the intriguing possibility that mycobacterial-specific ribosome proteins such as bS22 could facilitate leaderless translation initiation in mycobacteria (42).

Mammalian mitochondrial mRNAs are also leaderless and, although *S. cerevisiae* mitochondrial mRNAs have long 5′-UTR leaders, they lack Shine-Dalgarno sequences, and require transcript-specific translational activators to initiate translation (2). It is tempting to speculate that the acquisition of mS38 by mitochondrial ribosomes co-evolved with the loss of Shine-Dalgarno sequences in the mRNAs. However, it has been recently shown that in mammalian mitochondria, start codon selection could occur by mitoribosome-specific mRNA engagement and subsequent threading of the mRNA into the mRNA channel for start codon-anticodon interactions, which would be stabilized by a domain insertion that is present in the mammalian mitochondrial initiation factor 2 (44). Focusing on *S. cerevisiae*, although the 15S rRNA of yeast mitochondria lacks the Shine-Dalgarno sequence (45), it has been shown to have in its 3’ end a 10-nucleotide sequence complementary to different extents with sequences in the 5′ leaders of the messages for *COX1* (10 nt), *COX2* (7 nt), *COX3* (7 nt) and *ATP6* (8 nt) (45). However, yeast mitoribosome structural studies have shown that the 3′ end of the 15S rRNA is not constrained to a position at the mRNA channel exit but extends into the mtSSU body where it is surrounded by mitoribosomal proteins (8). Different from bacteria, a wide V-shaped canyon flanked by mitoribosomal proteins forms at the mRNA channel exit, above which mRNA-specific translational activators may functionally interact (8). The presence of mRNA occupying this canyon during translation initiation would explain ribosome-profiling data, which have shown that the yeast mitoribosome protects longer mRNA stretches during active translation than cytosolic ribosomes (∼38 versus 28 nucleotides) (3). The absence of mS38 in the deletion strain might change the flexibility of the elements forming the mRNA exit channel. Such a modification could affect the transit of specific mRNAs (e.g. *COX1, COX2* and *COX3*), perhaps giving them a chance to interact with the 3′ end of the 15S rRNA unproductively or merely misfold, thus jamming the channel.

In conclusion, we propose that mS38 is a core component of the mitoribosomal SSU that may exert regulatory effects by interacting with specific mRNAs and by interfacing with structural elements in the mRNA exit channel to facilitate their correct translation initiation. This hypothesis is reminiscent of the ‘ribosome filter’ model according to which specific riboproteins would allow the ribosome to interface with specific subsets of mRNAs to regulate their translation (46).

## Supplementary Material

gkz266_Supplemental_File
