# Supplementary material for: The mitoribosome-specific protein mS38 is preferentially required for synthesis of cytochrome c oxidase subunits
Source: Nucleic Acids Res. 2019 Apr 10;47(11):5746–60. doi: 10.1093/nar/gkz266 (PMC6582356; doi:10.1093/nar/gkz266)
Supplement: gkz266_Supplemental_File [file gkz266_supplemental_file.pdf]

SUPPLEMENTAL FIGURES

Figure S1. The rate of mitochondrial protein synthesis is strongly attenuated in Δms38 cells. Related to Figure 1.
Metabolic labeling with ^{35}S-methionine (10 μCi; 17.20 nM) of newly synthesized mitochondrial products in whole cells during increasing pulse times in the presence of cycloheximide to inhibit cytoplasmic protein synthesis. Immunoblotting for Porin was used as a loading control. Newly synthesized polypeptides are identified on the right. In the lower panel, raw densitometry values for the indicated individual proteins were plotted to visualize their synthesis kinetics.

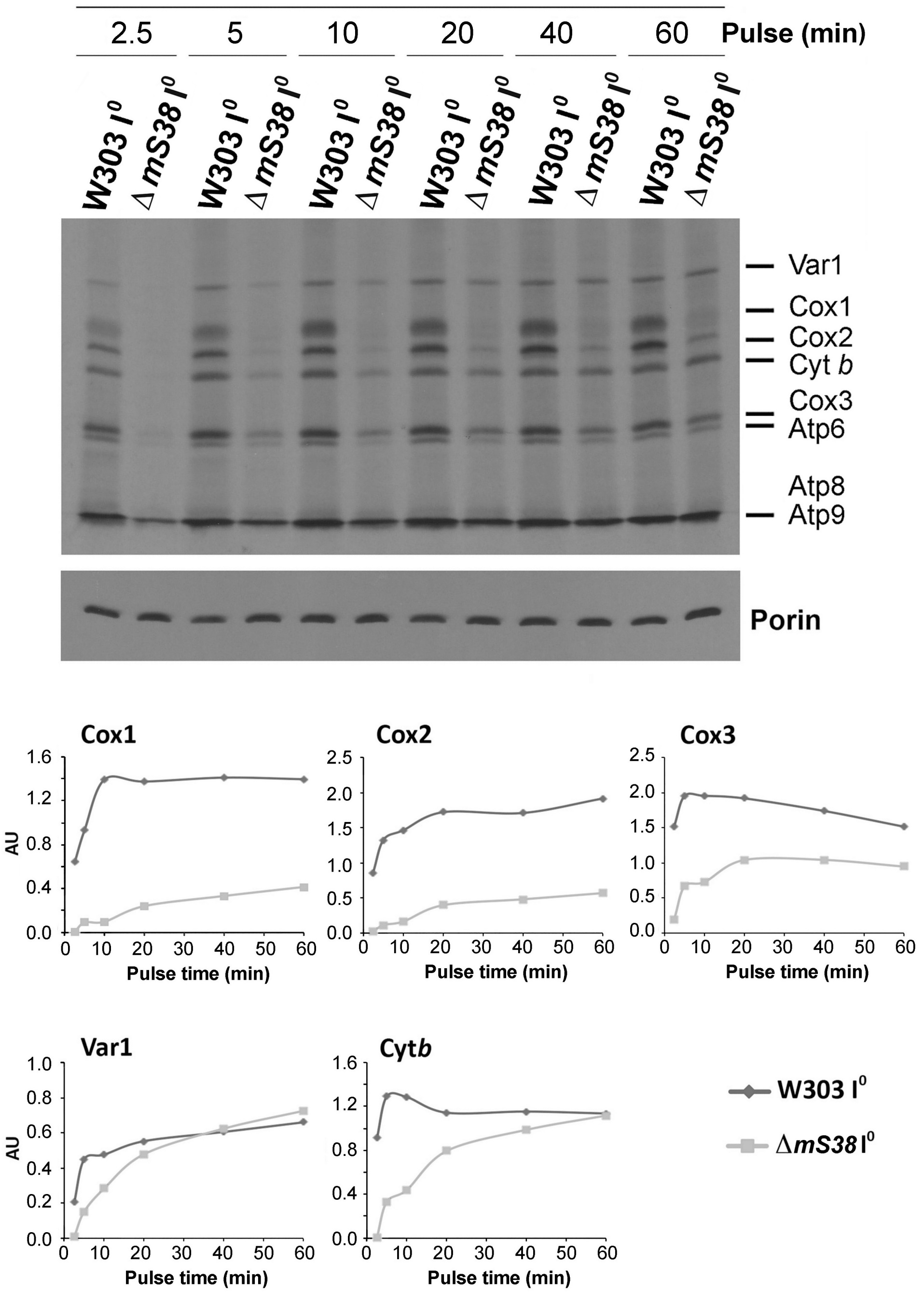

Figure S2. Mitochondrial protein synthesis and stability of newly synthesized polypeptides in strains overexpressing or deleted for mS38. Related to Figure 2.

In *vivo* mitochondrial protein synthesis following the incorporation of [^{35}S]-methionine into newly synthesized products in the presence of cycloheximide to inhibit cytoplasmic protein synthesis.

(A) The indicated strains were pulsed with [^{35}S]-methionine as in Fig S1 for 10 minutes followed by addition of puromycin and excess cold methionine and chased of the newly-synthesized mitochondrial polypeptides for the indicated times. Following SDS-PAGE, transfer to a nitrocellulose membrane and autoradiography, the signals were digitalized and quantified using Image J. The Cox1/Var1 signal ratio was plotted in the lower panel graph.

(B) Wild-type W303 carrying intron-less mtDNA (W303 I^0) transformed with an empty vector (EV) or a construct overexpressing mS38 pulsed with [^{35}S]-methionine during the indicated increasing times. Porin was used as a loading control.

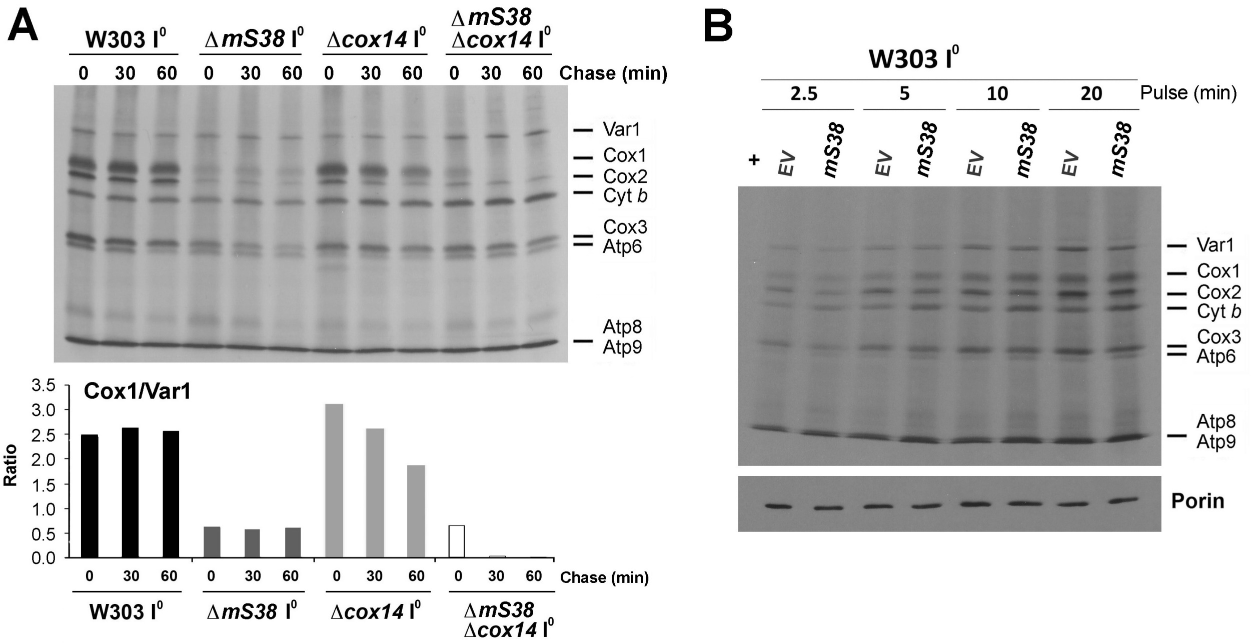

Figure S3. Detection of endogenous and functional GST-tagged mS38. Related to Figure 3.
(A) Generation of an antibody against mS38. Immunoblot analysis of mS38 steady-state levels in mitochondria isolated from WT, Δ*mS38* and the deletion strain overexpressing a hemagglutinin (HA)-tagged version of mS38, using a rabbit polyclonal antibody generated against purified recombinant mS38. Porin was used as a loading control. * indicates endogenous mS38 and ** indicates mS38-HA. Although mS38-HA is stable and overexpressed, only poorly complemented the Δ*mS38* strain (not shown) and therefore was not used for further experiments. (B) GST (Glutathione S-transferase)-tagged mS38 is a functional protein. The left panel presents a growth test using serial dilutions of the indicated strains in complete fermentable (YPD) or respiratory (YEPG) media. *mS38-GST* refers to the Δ*mS38* strain expressing in single copy the fusion protein. Pictures were taken after 2 days of growth at 30°C. (C) Steady-state levels of mS38-GST assessed by immunoblotting using and antibody against GST. Porin level was used as a loading control. (D) Mitochondrial protein synthesis in the indicated strains performed as in Figure S1 with a 10 min pulse.

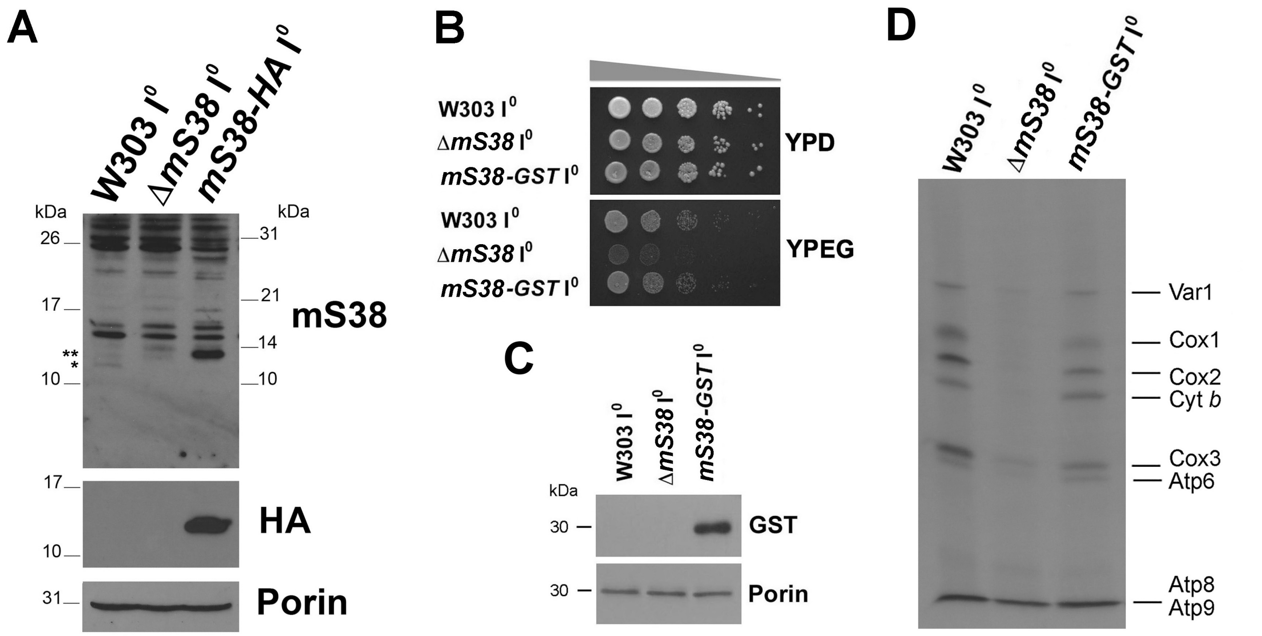

Figure S4. Deletion or overexpression of *PET54* does not bypass the requirement of mS38 for translation of *COX1* mRNA. Related to figure 5.

(A) FLAG-tagged Pet54 is a functional protein. Growth test using serial dilutions of the indicated strains in complete media containing fermentable (YPD) or respiratory (YEPG) carbon sources. *PET54-FLAG* refers to the Δ*pet54* strain expressing in single copy the fusion protein. Pictures were taken after 2 days of growth at 30°C.

(B) Expression of Pet54-FLAG was assessed by immunoblotting, using an antibody against Flag. Porin level was used as a loading control.

(C) Growth test using serial dilutions of the indicated strains in complete fermentable (YPD) or respiratory (YEPG) media. Pictures were taken after 2 days of growth at 30°C.

(D) Endogenous cell respiration of the indicated strains grown in minimum synthetic media containing galactose as the carbon source. Oxygen consumption was measured polarographically and the results are expressed as percentage of wild-type. The bars represent the average of three independent repetitions +/- SD.

(E) In *vivo* mitochondrial protein synthesis following the incorporation of [^{35}S]-methionine into newly synthesized products in the presence of cycloheximide to inhibit cytoplasmic protein synthesis, performed as in Fig S1, using the indicated strains. Porin was used as a loading control. The lower panel shows a quantification of the Cox1 signal *vs* Porin.

(F) Growth test using serial dilutions of the indicated strains in complete fermentable (YPD) or respiratory (YEPG) media. Pictures were taken after 2 days of growth at 30°C.

(G) In *vivo* mitochondrial protein synthesis performed as in panel (E), using the indicated strains.

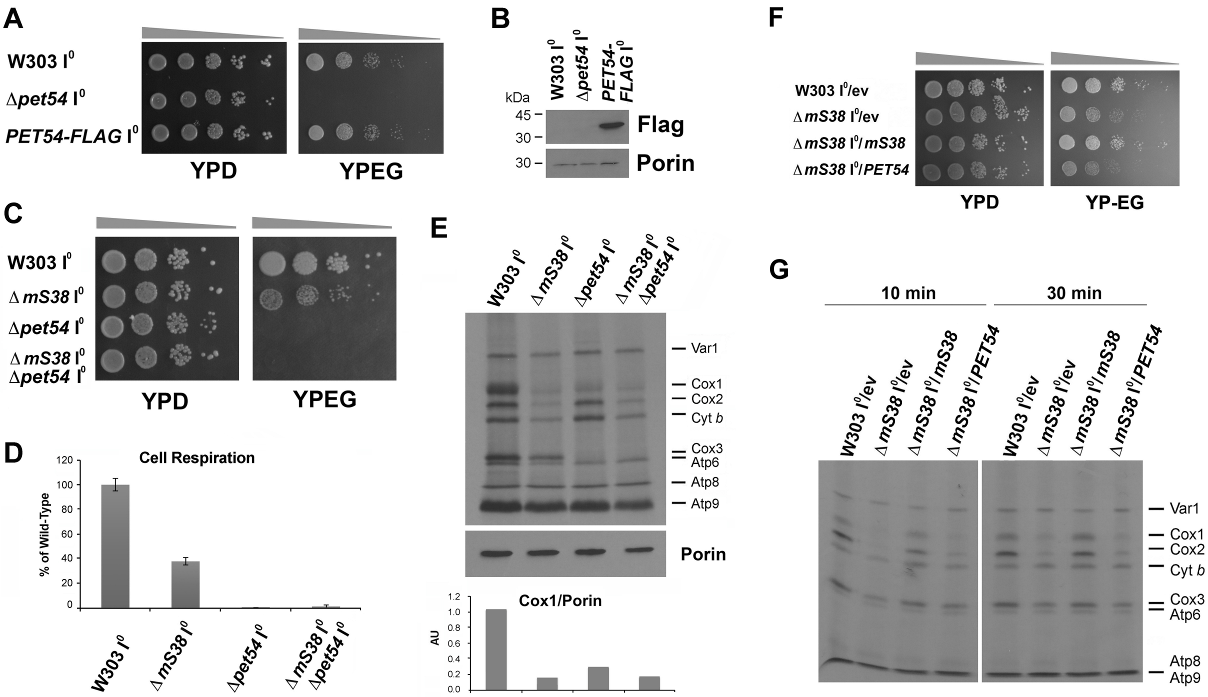

Figure S5. Overexpression of *MAM33* does not bypass the requirement of mS38 for translation of *COX1* mRNA. Related to figure 5.
(A) Expression of Mam33-FLAG was assessed by immunoblotting, using an antibody against Flag. Porin level was used as a loading control.
(B) FLAG-tagged Mam33 is a functional protein. In *vivo* mitochondrial protein synthesis following the incorporation of [^{35}S]-methionine into newly synthesized products in the presence of cycloheximide to inhibit cytoplasmic protein synthesis, performed as in Fig S1, using the indicated strains. Porin was used as a loading control.
(C) Growth test using serial dilutions of the indicated strains in complete fermentable (YPD) or respiratory (YEPG) media. Pictures were taken after 2 days of growth at 30°C.

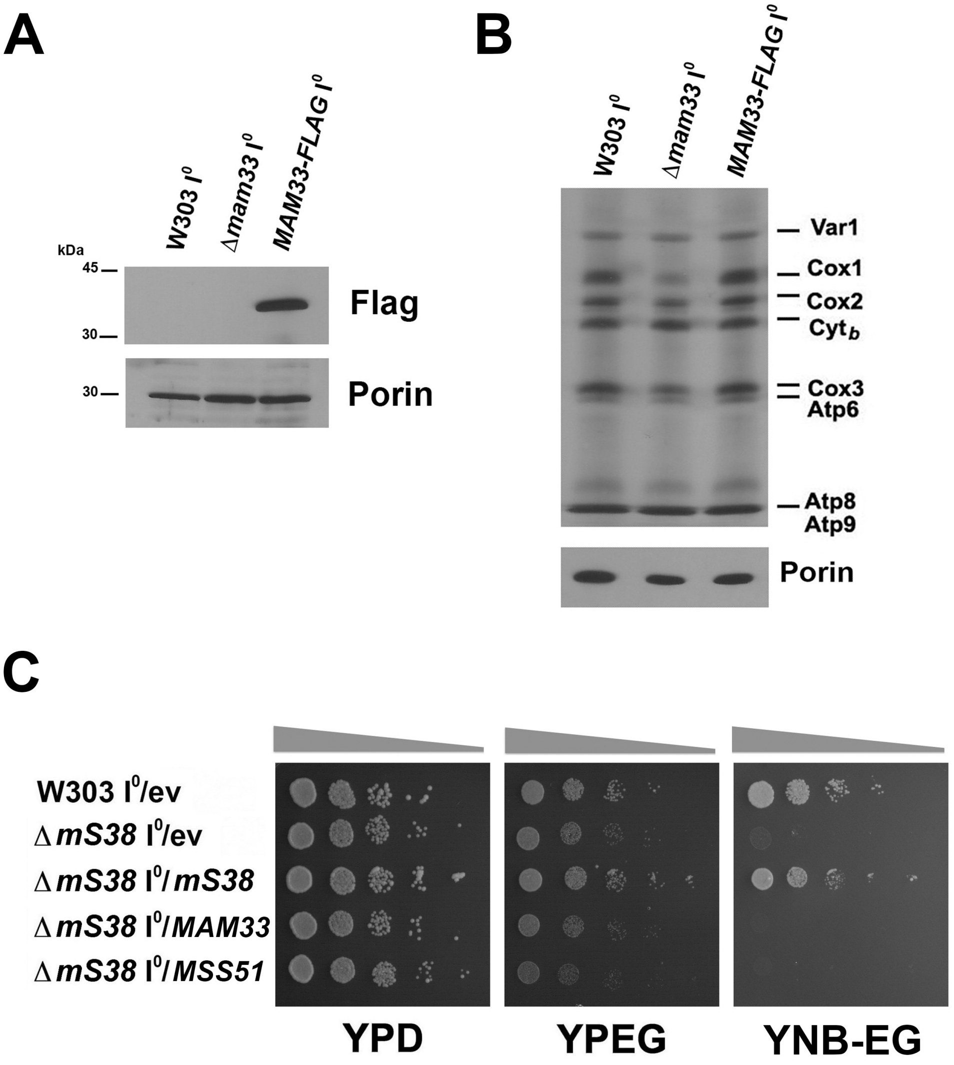

Figure S6. The paromomycin-resistance mutation interacts with the *mS38* null mutation. Related to figure 6.
In vivo mitochondrial protein synthesis on the indicated strains following incorporation of [^{35}S]-methionine into newly synthesized polypeptides during 10 min, in the presence of cycloheximide to inhibit cytoplasmic protein synthesis, performed as in Fig S1. The WT (+mS38) and null mutant of *mS38* (Δ) strains were created in mtDNA backgrounds carrying paromomycin sensitive (Par^S) or resistant (Par^R) 15S rRNA alleles. Porin was used as a loading control.

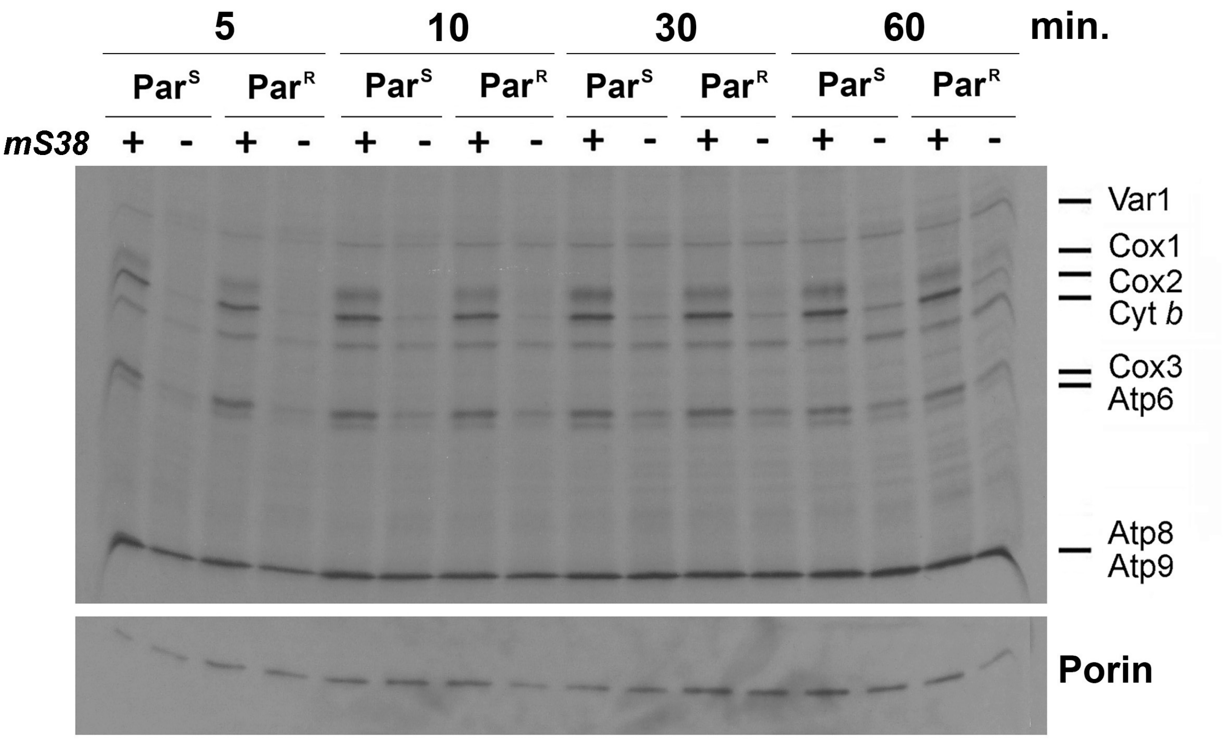

Figure S7. Functional equivalence of *S. cerevisiae* mS38 with mS38 homologs across evolution. Related to figure 7.
(A-C) Growth test using serial dilutions of the indicated strains in complete fermentable (YPD) or respiratory (YEPG) media, or in synthetic yeast nitrogen base supplemented with prototrophic requirements and respiratory carbon sources (YNB-EG). Pictures were taken after 2 days of growth at 30°C. *Sc, Saccharomyces cerevisiae; Kl, Kluyveromyces lactis; Yl, Yarrowia lipolytica; At, Arabidopsis thaliana; Os, Oriza sativa; Hs, Homo sapiens.* hMRPS38 is the human homologue of *S. cerevisiae* mS38.
(D) Stability of newly synthesized mitochondrial polypeptides assessed by *in vivo* mitochondrial protein synthesis. The indicated strains were pulsed with [^{35}S]-methionine for 10 minutes in the presence of cycloheximide to inhibit cytoplasmic protein synthesis followed by addition of puromycin and excess cold methionine and chased of the newly-synthesized mitochondrial polypeptides for the indicated times.

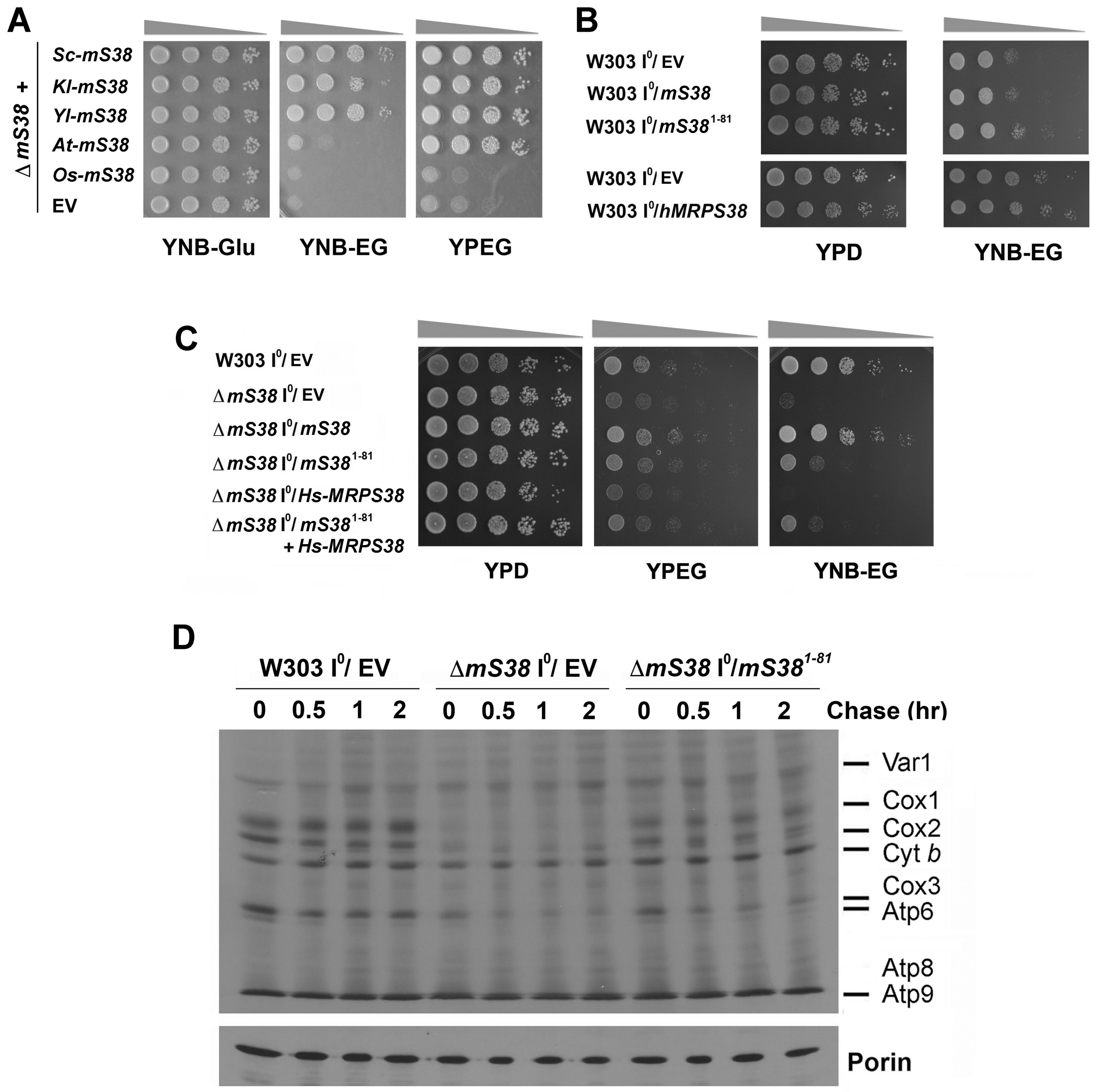

Figure S8. Modeling of mS38 structure. Related to figure 7.
Structure of *S. cerevisiae* mS38 as obtained by modeling the whole mature protein (aa 16-111) using Quark (server https://zhanglab.ccmb.med.umich.edu/QUARK2/)(1). According to the server, “QUARK is a computer algorithm for *ab initio* protein structure prediction and protein peptide folding, which aims to construct the correct protein 3D model from amino acid sequence only. QUARK models are built from small fragments (1-20 residues long) by replica-exchange Monte Carlo simulation under the guide of an atomic-level knowledge-based force field.” (A) Predicted secondary structure and solvent accessibility. (B) Top five structure models. Key amino acid residues are indicated in model #1.

A

Predicted Secondary Structure

```
                          20                  40                  60                  80
                           |                   |                   |                   |
Sequence     TVVKKPSCGSYFNRTFQTAINTTMPPMQEGMLSTMMMMTATATRITGTVSEPLNGSNIVMQLDSVMRKRKKKMKKHKLRKRRKREKAERRKLSQGR
Prediction   CCCCCCCHHHHHHHHHHHHHCCCCCHHHHHHHHHHHHHHHHHHHCCSSCCCCCCCCSSSHHHHHHHHHHHHHHHHHHHHHHHHHHHHHHHHCCCC
Conf. Score  97768977999979999987689986899999999999999988645347899998663337999999999998999999999999888876455799

             H:Helix; S:Strand; C:Coil
```

Predicted Solvent Accessibility

```
                          20                  40                  60                  80
                           |                   |                   |                   |
Sequence     TVVKKPSCGSYFNRTFQTAINTTMPPMQEGMLSTMMMMTATATRITGTVSEPLNGSNIVMQLDSVMRKRKKKMKKHKLRKRRKREKAERRKLSQGR
Conf. Score  55343220220012001101222112122000100000101013021202221312100010121023333323433133333333233423334
             Values range from 0 (buried residue) to 9 (highly exposed residue)
```

B

Top 5 Final Structure Models

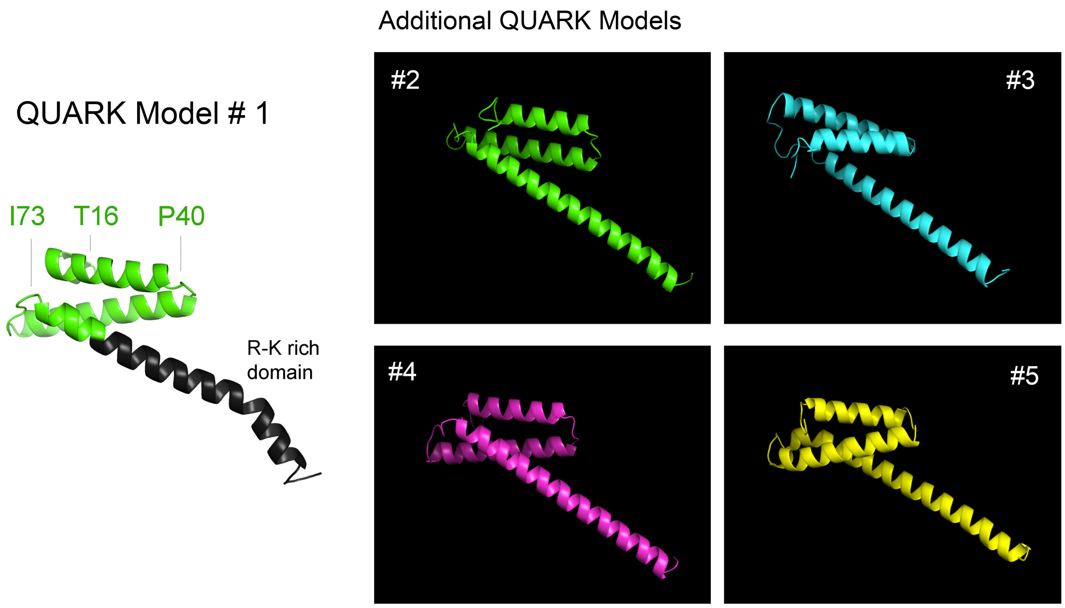

SUPPLEMENTAL TABLES

Table S1. Genotype and source of *S. cerevisiae* strains used.

Strain	Genotype	Source
Strains in the W303 background		
αW303-1B / I^0	*MATα, ade2-1 his3-1,15 leu2-3,112 trp1-1 ura3-1,* ρ^+ I^0	(2)
aW303-1A / I^0	*MATa, ade2-1 his3-1,15 leu2-3,112 trp1-1 ura3-1,* ρ^+ I^0	(2)
aW303-1A / I^0 Δ*mS38*	*MATa, ade2-1 his3-1,15 leu2-3,112 trp1-1 ura3-1 ΔmS38::HIS3*	(3)
aW303-1A / I^0 Δ*mS38* + *mS38*-GST	*MATa, ade2-1 his3-1,15 leu2-3,112 trp1-1 ura3-1 ΔmS38::HIS3 + YIplac128- mS38-GST,* ρ^+ I^0	This study
aW303-1A / I^0 + YIplac128	*MATa, ade2-1 his3-1,15 leu2-3,112 trp1-1 ura3-1, + YIplac128,* ρ^+ I^0	This study
aW303-1A / I^0 + *mS38*$^{1-81}$	*MATa, ade2-1 his3-1,15 leu2-3,112 trp1-1 ura3-1, + YIplac128- mS38*$^{1-81}$, ρ^+ I^0	This study
aW303-1A / I^0 Δ*mS38* + YIplac128	*MATa, ade2-1 his3-1,15 leu2-3,112 trp1-1 ura3-1 ΔmS38:: HIS3, + YIplac128,* ρ^+ I^0	This study
aW303-1A / I^0 Δ*mS38* + *mS38*$^{1-81}$	*MATa, ade2-1 his3-1,15 leu2-3,112 trp1-1 ura3-1 ΔmS38:: HIS3, + YIplac128- mS38*$^{1-81}$, ρ^+ I^0	This study
aW303-1A / I^0 Δ*mS38* + *mS38*$^{81-111}$	*MATa, ade2-1 his3-1,15 leu2-3,112 trp1-1 ura3-1 ΔmS38:: HIS3, + YIplac128- mS38*$^{81-111}$, ρ^+ I^0	This study
aW303-1A / I^0 Δ*mS38* + *mS38*$^{81-111}$*-GST*	*MATa, ade2-1 his3-1,15 leu2-3,112 trp1-1 ura3-1 ΔmS38:: HIS3, + YIplac128- mS38*$^{81-111}$*-GST,* ρ^+ I^0	This study
aW303-1A / I^0 Δ*mS38* + *COX4*^{TS}*-mS38*$^{81-111}$	*MATa, ade2-1 his3-1,15 leu2-3,112 trp1-1 ura3-1 ΔmS38:: HIS3, + YIplac128- COX4*^{TS}*-mS38*$^{81-111}$, ρ^+ I^0	This study
aW303-1A / I^0 + YEplac112	*MATa, ade2-1 his3-1,15 leu2-3,112 trp1-1 ura3-1 + YEplac112,* ρ^+ I^0	This study
aW303-1A / I^0 + *TEF1p-hMRPS38*	*MATa, ade2-1 his3-1,15 leu2-3,112 trp1-1 ura3-1 + YEplac112- TEF1p-hMRPS38,* ρ^+ I^0	This study
aW303-1A / I^0 Δ*mS38* + YEplac112	*MATa, ade2-1 his3-1,15 leu2-3,112 trp1-1 ura3-1 ΔmS38:: HIS3, + YEplac112,* ρ^+ I^0	This study
aW303-1A / I^0 Δ*mS38* + *TEF1p-hMRPS38*	*MATa, ade2-1 his3-1,15 leu2-3,112 trp1-1 ura3-1 ΔmS38:: HIS3, + YEplac112- TEF1p-hMRPS38,* ρ^+ I^0	This study
aW303-1A / I^0 Δ*mS38* + *mS38*$^{1-81}$*, TEF1p-hMRPS38*	*MATa, ade2-1 his3-1,15 leu2-3,112 trp1-1 ura3-1 ΔmS38:: HIS3, + YIplac128- mS38*$^{1-81}$ *YEplac112- TEF1p-hMRPS38,* ρ^+ I^0	This study
aW303-1A / I^0 Δ*pet54*	*MATa, ade2-1 his3-1,15 leu2-3,112 trp1-1 ura3-1 Δpet54:: KanMX*, ρ^+ I^0	(4)
aW303-1A / I^0 Δ*pet54* + *PET54-FLAG*	*MATa, ade2-1 his3-1,15 leu2-3,112 trp1-1 ura3-1 Δpet54:: KanMX, + YIplac204-PET54-FLAG,* ρ^+ I^0	This study
aW303-1A / I^0 Δ*mS38*Δ*pet54*	*MATa, ade2-1 his3-1,15 leu2-3,112 trp1-1 ura3-1 ΔmS38:: HIS3 Δpet54:: KanMX* , ρ^+ I^0	This study
aW303-1A / I^0 Δ*mS38*Δ*pet54* + *PET54-FLAG, mS38-GST*	*MATa, ade2-1 his3-1,15 leu2-3,112 trp1-1 ura3-1 ΔmS38:: HIS3 Δpet54:: KanMX + YIplac204-PET54-FLAG YIplac128- mS38-GST,* ρ^+ I^0	This study

aW303-1A / I^0 Δ*mS38*Δ*pet54* + *PET54-FLAG*	*MATa, ade2-1 his3-1,15 leu2-3,112 trp1-1 ura3-1* Δ*mS38:: HIS3* Δ*pet54:: KanMX + YIplac204-PET54-FLAG,* ρ^+ I^0	This study
aW303-1A / I^0 Δ*mS38* + *PET54*	*MATa, ade2-1 his3-1,15 leu2-3,112 trp1-1 ura3-1* Δ*mS38:: HIS3 + YEplac181-PET54,* ρ^+ I^0	This study
aW303-1A / I^0 Δ*mam33*	*MATa, ade2-1 his3-1,15 leu2-3,112 trp1-1 ura3-1* Δ*mam33:: KanMX* ρ^+ I^0	This study
aW303-1A / I^0 Δ*mam33* + *MAM33-FLAG*	*MATa, ade2-1 his3-1,15 leu2-3,112 trp1-1 ura3-1* Δ*mam33:: KanMX + YIplac204-MAM33-FLAG* ρ^+ I^0	This study
aW303-1A / I^0 Δ*mam33*Δ*mS38* + *MAM33-FLAG*	*MATa, ade2-1 his3-1,15 leu2-3,112 trp1-1 ura3-1* Δ*mS38:: HIS3* Δ*mam33:: KanMX + YIplac204-MAM33-FLAG* ρ^+ I^0	This study
aW303-1A / I^0 Δ*mS38* + *MAM33*	*MATa, ade2-1 his3-1,15 leu2-3,112 trp1-1 ura3-1* Δ*mS38:: HIS3 + YEplac181-MAM33-FLAG* ρ^+ I^0	This study
αW303-1A / I^0 Δ*pet309* + *PET309-HA*	*MATα , ade2-1 his3-1,15 leu2-3,112 trp1-1 ura3-1, Δpet309::HIS3 + YIplac211-PET309-HA,* ρ+ I^0	(5)
αW303-1A / I^0 Δ*mS38*Δ*pet309* + *PET309-HA*	*MATα , ade2-1 his3-1,15 leu2-3,112 trp1-1 ura3-1,* Δ*mS38:: KanMX Δpet309::HIS3 + YIplac211-PET309-HA,* ρ+ I^0	This study
aW303-1A / I^0 + YEp351	*MATa, ade2-1 his3-1,15 leu2-3,112 trp1-1 ura3-1* + YEp351, ρ^+ I^0	This study
aW303-1A / I^0 + *MSS51*	*MATa, ade2-1 his3-1,15 leu2-3,112 trp1-1 ura3-1* + YEp351-MSS51, ρ^+ I^0	This study
aW303-1A / I^0 Δ*mS38* + YEp351	*MATa, ade2-1 his3-1,15 leu2-3,112 trp1-1 ura3-1* Δ*mS38:: HIS3* + YEp351, ρ^+ I^0	This study
aW303-1A / I^0 Δ*mS38* + *MSS51*	*MATa, ade2-1 his3-1,15 leu2-3,112 trp1-1 ura3-1* Δ*mS38:: HIS3* + YEp351-*MSS51,* ρ^+ I^0	This study
aW303-1A / I^0 + YIplac211	*MATa, ade2-1 his3-1,15 leu2-3,112 trp1-1 ura3-1* + YIplac211, ρ^+ I^0	This study
aW303-1A / I^0 + *mss51*^{F199I}	*MATa, ade2-1 his3-1,15 leu2-3,112 trp1-1 ura3-1* + YIplac211-*mss51*^{F199I}, ρ^+ I^0	This study
aW303-1A / I^0 Δ*mS38* + YIplac211	*MATa, ade2-1 his3-1,15 leu2-3,112 trp1-1 ura3-1 mS38:: HIS3* + YIplac211, ρ^+ I^0	This study
aW303-1A / I^0 Δ*mS38* + *mss51*^{F199I}	*MATa, ade2-1 his3-1,15 leu2-3,112 trp1-1 ura3-1 mS38:: HIS3* + YIplac211-*mss51*^{F199I}, ρ^+ I^0	This study
aW303-1A / I^0 + YEplac181	*MATa, ade2-1 his3-1,15 leu2-3,112 trp1-1 ura3-1* + YEplac181, ρ^+ I^0	This study
aW303-1A / I^0 + *mS38*-YEplac181	*MATa, ade2-1 his3-1,15 leu2-3,112 trp1-1 ura3-1* + YEplac181-*mS38,* ρ^+ I^0	This study
aW303-1A / I^0 Δ*mS38* + *mS38*-YEplac181	*MATa, ade2-1 his3-1,15 leu2-3,112 trp1-1 ura3-1* Δ*mS38:: HIS3* + YEplac181-*mS38,* ρ^+ I^0	This study
aW303-1A / I^0 Δ*mS38* + *PET54*-YEplac181	*MATa, ade2-1 his3-1,15 leu2-3,112 trp1-1 ura3-1* Δ*mS38:: HIS3* + YEplac181-*PET54,* ρ^+ I^0	This study
aW303-1A / I^0 Δ*cox14*	*MATa, ade2-1 his3-1,15 leu2-3,112 trp1-1 ura3-1* Δ*cox14::TRP1,* ρ^+ I^0	(6)
aW303-1A / I^0 Δ*mS38*Δ*cox14*	*MATa, ade2-1 his3-1,15 leu2-3,112 trp1-1 ura3-1* Δ*mS38::HIS3* Δ*cox14::TRP1,* ρ^+ I^0	(3)
αW303-1A / I^0 Δ*shy1*	*MATα, ade2-1 his3-1,15 leu2-3,112 trp1-1 ura3-1* Δ*shy1::URA3,* ρ^+ I^0	(7)

αW303-1A / I^0 Δ*shy1*Δ*cox14*	*MATα, ade2-1 his3-1,15 leu2-3,112 trp1-1 ura3-1 Δshy1::URA3 Δcox14::TRP1,* ρ$^+$ I^0	(6)
aW303-1A / I^0 Δ*cox11*	*MATa, ade2-1 his3-1,15 leu2-3,112 trp1-1 ura3-1 Δcox11::HIS3,* ρ$^+$ I^0	(8)
aW303-1A / I^0 Δ*mS38*Δ*cox11*	*MATa, ade2-1 his3-1,15 leu2-3,112 trp1-1 ura3-1 Δcox11::HIS3 ΔmS38::KanMX,* ρ$^+$ I^0	This study
aW303-1A / I^0 Δ*mS38*Δ*cox11 + mS38-GST*	*MATa, ade2-1 his3-1,15 leu2-3,112 trp1-1 ura3-1 Δcox11::HIS3 ΔmS38::KanMX + YIplac128- mS38-GST,* ρ$^+$ I^0	This study
aW303-1A / I^0 Δ*nuc1*	*MATa, ade2-1 his3-1,15 leu2-3,112 trp1-1 ura3-1 Δnuc1::KanMX,* ρ$^+$ I^0	This study
aW303-1A / I^0 Δ*nuc1*Δ*mS38*	*MATa, ade2-1 his3-1,15 leu2-3,112 trp1-1 ura3-1 Δnuc1::KanMX ΔmS38::HIS3,* ρ$^+$ I^0	This study
aW303-1A / I^0 Par^R	*MATa, ade2-1 his3-1,15 leu2-3,112 trp1-1 ura3-1,* ρ$^+$ I^0 15S-Par^R	This study
aW303-1A / I^0 Δ*mS38* Par^R	*MATa, ade2-1 his3-1,15 leu2-3,112 trp1-1 ura3-1, ΔmS38:: KanMX ,* ρ$^+$ I^0 15S-Par^R	This study
aW303-1A / I^0 Δ*mss51 + mss51*^{F199I}	*MATa, ade2-1 his3-1,15 leu2-3,112 trp1-1 ura3-1, Δmss51:: HIS3 + YIp351-mss51*^{F199I}, ρ$^+$ I^0	This study
aW303-1A / I^0 Δ*mss51 + mss51*^{F199I} Par^R	*MATa, ade2-1 his3-1,15 leu2-3,112 trp1-1 ura3-1, Δmss51:: HIS3 + YIp351-mss51*^{F199I} , ρ$^+$ I^0 15S-Par^R	This study
aW303-1A / Δ*mS38 + Kl-mS38*	*MATa, ade2-1 his3-1,15 leu2-3,112 trp1-1 ura3-1, ΔmS38::HIS3, + pDB20- Kl-mS38,* ρ$^+$	This study
aW303-1A / Δ*mS38 + Yl-mS38*	*MATa, ade2-1 his3-1,15 leu2-3,112 trp1-1 ura3-1, ΔmS38::HIS3, + pDB20- Yl-mS38,* ρ$^+$	This study
aW303-1A / Δ*mS38 + At-mS38*	*MATa, ade2-1 his3-1,15 leu2-3,112 trp1-1 ura3-1, ΔmS38::HIS3, + pDB20-At-mS38,* ρ$^+$	This study
aW303-1A / Δ*mS38 + Os-mS38*	*MATa, ade2-1 his3-1,15 leu2-3,112 trp1-1 ura3-1, ΔmS38::HIS3, + pDB20-Os-mS38,* ρ$^+$	This study
YC162	*MATα, ade2-1 his3-1,15 leu2-3,112 trp1-1 ura3-1 Δarg8::KanMX [ρ+, cox1Δ::ARG8*^{m}*]*	This study
RGV139	*MAT a, his3-1,15 leu2-3,112 trp1-1 ura3-1 Δarg8::KanMX [ρ+, cox2Δ::ARG8*^{m}*]*	This study
RGV140	*MAT a, his3-1,15 leu2-3,112 trp1-1 ura3-1 Δarg8::KanMX [ρ+, cox3Δ::ARG8*^{m}*]*	This study
RGV137	*MATα, ade2-1 his3-1,15 leu2-3,112 trp1-1 ura3-1 Δarg8::KanMX [ρ+, cobΔ::ARG8*^{m}*]*	(12), This study
RGV141	*MATα, ade2-1 his3-1,15 leu2-3,112 trp1-1 ura3-1 Δarg8::KanMX, ΔmS38:: HIS3 [ρ+, cox1Δ::ARG8*^{m} *]*	This study
RGV142	*MAT a, his3-1,15 leu2-3,112 trp1-1 ura3-1 Δarg8::KanMX ΔmS38:: HIS3 [ρ+, cox2Δ::ARG8*^{m}*]*	This study
RGV143	*MAT a, his3-1,15 leu2-3,112 trp1-1 ura3-1 Δarg8::KanMX ΔmS38:: HIS3 [ρ+, cox3Δ::ARG8*^{m}*]*	This study
RGV145	*MATα, ade2-1 his3-1,15 leu2-3,112 trp1-1 ura3-1 Δarg8::KanMX ΔmS38:: HIS3 [ρ+, cobΔ::ARG8*^{m}*]*	This study

Table S2. Antibodies used. The list of antibodies against *S. cerevisiae* mitoribosomal proteins includes double nomenclature to avoid confusion.

Protein	Company/Reference
OXPHOS subunits and assembly fators	
Cox1	Anti-MTCO1 antibody [11D8B7] - Abcam cat# 110270
Cox2	Anti-MTCO2 antibody [4B12A5] - Abcam cat# 110271
Cox3	Anti-MTCO3 antibody [DA5BC4] - Abcam cat# 110259
Cox4	Anti-Cytochrome *c* oxidase subunit 4 [1A12A12] - Abcam cat# ab110272
Cyt*b*	Gift from Dr. A. Tzagoloff
Rip1	Gift from Dr. R. Stuart
Atp2	Gift from Dr. S. Ackerman
Sdh2	Gift from Dr. D. Winge
Mss51	Reference (7)
Cox14	Reference (13)
Coa3	Reference (14)
Mss116	Reference (5)
Mitoribosome subunits	
uL24	Gift from Dr. R. Stuart
mS37	Gift from Dr. A. Tzagoloff
mS38	This study
uL2	Reference (15)
uL16	Reference (5)
bL32	Gift from Dr. R. Stuart
bL33	Reference (5)
bS1	Gift from Dr. M. Ott
mS38	This study
Protein tags	
GST	Sigma cat# G1160
HA	Invitrogen cat# 71-5500
FLAG	Sigma cat# F1804
Loading controls	
Porin	Anti-VDAC1/Porin antibody [16G9E6BC4] - Abcam cat# 110326
Cit1	Gift from Dr. TD. Fox
Others	
Arg8	Gift from Dr. TD. Fox

Table S3. Mitoribosome small subunit (mtSSU) proteins identified by mass spectrometry in sucrose gradient fractions from wild-type and Δ*mS38* mitochondrial extracts. Related to Figure 3.

SSU subunits		Spectral Count	
New name	Old name	W303 I^0	Δ*mS38* I^0
bS1	Mrp51	163	167
uS2	Mrp4	108	114
uS3	Var1	162	143
bS4	Nam9	132	117
uS5	Mrps5	63	59
bS6	Mrp17	33	40
uS7	Rsm7	80	69
uS8	Mrps8	89	77
uS9	Mrps9	100	96
uS10	Rsm10	67	63
uS11	Mrps18	35	35
uS12	Mrps12	19	20
uS13	Sws2	40	24
uS14	Mrp2	30	31
uS15	Mrps28	92	93
bS16	Mrps16	25	33
uS17	Mrps17	93	75
bS18c	Rsm18	20	21
uS19	Rsm19	9	10
bS21	Mrp21	37	34
mS23	Rsm25	156	129
mS26	Pet123	69	73
mS29	Rsm23	153	173
mS33	Rsm27	20	18
mS35	Rsm24	151	115
mS36	Ymr31	3	3
mS37	Mrp10	7	7
mS41	Fyv4	14	13
mS42	Rsm26	109	114
mS43	Mrp1	107	110
mS44	Mrp13	103	102
mS45	Mrps35	114	116
mS46	Rsm28	83	69
mS47	Ehd3	ND	ND
mS48	Ppe1	ND	ND
mS38	Cox24	ND	ND

SUPPLEMENTAL EXPERIMENTAL PROCEDURES

Yeast Strains and Media

All *S. cerevisiae* strains used are listed in Supplementary Table S1. Yeast cells were grown in the following standard culture media: YPD (2% glucose, 1% yeast extract, 2% peptone), YPGal (2% galactose, 1% yeast extract, 2% peptone), YPEG (2% ethanol, 3% glycerol, 1% yeast extract, 2% peptone), WO-EG (2% ethanol, 3% glycerol, 0.67% yeast nitrogen base), WO-Gal (2% galactose, 0.67% yeast nitrogen base). Strains grown in liquid and solid media were incubated at 30^oC unless otherwise indicated.

Sucrose Gradients
The sedimentation properties in sucrose gradients of mS38, other mitoribosomal proteins and translational activators from total mitochondrial extracts were analyzed essentially as described (16). Mitochondria were prepared by the method of Herrmann et al. (17). Four mg of protein were solubilized in 400 µl of extraction buffer (20 mM HEPES, pH 7.4, 0.5 mM PMSF, 0.8% digitonin, 0.5 mM $MgCl_2$ or 5 mM EDTA and 25 mM KCl) on ice for 5 min. EDTA was used to analyzed the 54S and 37S subunits separately, while Mg^{2+} was added to preserve their interaction into the 74S monosome. The clarified extract obtained by centrifugation at 50,000 × g for 15 min was applied to a 5 ml of linear 0.3 M–1.0 M sucrose gradient containing 20 mM HEPES, 0.5 mM PMSF, 0.1% digitonin, 0.5 mM $MgCl_2$ or 5 mM EDTA and 25 mM KCl. Following centrifugation for 3 h and 10 min at 40,000 r.p.m. using a Beckman 55Ti rotor, the gradients were collected in 14 equal fractions. Forty µl from each fraction was used to determine the distributions of the proteins of interest by immunoblot blot analysis. For some experiments the mitochondrial extracts were incubated with 600U/ml RNase (Fermentas) for 30 minutes on ice prior to loading onto the sucrose gradients.

Similarly, the sedimentation in sucrose gradient properties of Mss51 were analyzed as previously described (18). Four mg of protein from isolated mitochondria were solubilized in 400 µl of extraction buffer (20 mM HEPES, pH 7.4, 0.5 mM PMSF, 0.8% digitonin, 1.2 mM $MgCl_2$ and 150 mM KCl) on ice for 5 min. The clarified extract obtained by centrifugation at 50,000 × g for 15 min was applied to a 5 ml of linear 7% - 20% sucrose gradient containing 20 mM HEPES, 0.5 mM PMSF, 0.1% digitonin, 1.2 mM $MgCl_2$ and 150 mM KCl. Gradients were calibrated with hemoglobin (Hb) and lactate dehydrogenase (LDH). Following centrifugation for 12 h at 28,000 r.p.m. using a Beckman 55Ti rotor, the gradients were collected in 14 equal fractions.
All of the gradients were performed at least in triplicate using independent mitochondrial preparations. The gradients reported are representative of each strain because the patterns observed were reproducible.

For protein identification, protein from gradient fractions were methanol/chloroform precipitated as reported (15) and mass-spectrometry analysis was performed by SPARC BioCentre Molecular Analysis, The Hospital for Sick Children, Toronto, Canada.

RNA isolation and analysis
Total RNA was prepared from whole cells by a modified extraction method with hot-acidic phenol (19) and used for quantitative RT-qPCR analysis. Total RNA was also isolated from sucrose gradient fractions using Qiazol and miRNeasy mini kit (Qiagen) following the manufacturer's instructions. For quantitative RT-PCR analysis of mitochondrial RNAs, total RNA were treated with DNase I to remove mtDNA. cDNA was prepared using ThermoScript reverse transcriptase (Invitrogen) and random hexamers and used as a template in the subsequent PCR that was performed using Platinum UDG SYBR Green mastermix (Invitrogen) in a CFX96 Touch™ Real-Time PCR Detection System (Bio-Rad). Quantitative Real-Time PCR was performed according to Minimum Information for Publication of Quantitative Real-Time PCR Experiments (MIQE) guidelines (20). A standard curve was generated for each pair of primers and efficiency was measured between 90%-110%

Mitochondrial RNAs were quantified using triplicate cDNA samples and specific primer pairs for each gene following standard procedures.
Primers used in this study were:
COX1: 5'-TGATCAATTTTCATTACAGCGTT-3' and 5'-GGGTCACCACCTCCTGATAC-3'
COX2: 5'-CATGATTTTGCTATTCCAAG-3' and 5'-CATGCTCCATAGAAGACACC-3'

COB: 5'-TGGACAGATGTCACATTGAGG-3' and 5'-TGAGAACCCACCTCATAATCA-3'
21S rRNA: 5'-CCGAAAGCAAACGATCTAACT-3' and 5'-GCAAACCAGATTTGTCTTTCAC-3
15S rRNA: 5'-AATATTTGTGCCAGCAGTCG-3' and 5'-CGGATCCTTTAAACCATTATG-3'

The calculations of rRNA and mRNA levels in each sucrose gradient fraction were performed using the cycle threshold (CT) values (also known as *quantification cycle* (C_q), according to the RDML (Real-Time PCR Data Markup Language) data standard (21)), and the ΔΔCt method (22). The abundance of each transcript in each fraction of a given gradient was expressed as percentage of the sum of the abundance in all fractions of that gradient as reported (5,23).

PET54-FLAG immunoprecipitation
Mitochondrial extracts from mitochondria (1 mg) isolated from the indicated strains were prepared in the presence of 0.8% digitonin, 20 mM Hepes, 25 mM KCl, 0.5 mM $MgCl_2$ and 0.1 mM PMSF. The extracts were centrifuged at 25,000 g at 4^oC for 15 min, and precleared extracts were used in the immunoprecipitation experiment with FLAG-antibody or protein A conjugated agarose beads, as indicated. The immunoprecipitation reaction was performed at 4^0C temperature for 4 h. Following the immunoprecipitation reaction, the beads were washed five times with extraction buffer and resuspended in Laemmli buffer (24). Fractions of the input, unbound and bound material were analyzed by immunoblotting.

Miscellaneous Procedures
Standard procedures were used for the preparation and ligation of DNA fragments, and for transformation and recovery of plasmid DNA from *E. coli* (25). Yeast were transformed as described (26). Protein concentration was measured with the Folin phenol reagent (27). Proteins were separated by SDS-PAGE in the buffer system of Laemmli (24), and membranes with immobilized proteins were treated with antibodies against the appropriate proteins followed by a second reaction with anti-mouse or anti-rabbit IgG conjugated to horseradish peroxidase (Sigma, St. Louis, MO). The Super Signal chemiluminescent substrate kit (Pierce, Rockford, IL) was used for the final detection.

SUPPLEMENTAL REFERENCES

1. Xu, D. and Zhang, Y. (2012) Ab initio protein structure assembly using continuous structure fragments and optimized knowledge-based force field. *Proteins*, 80, 1715-1735.
2. Zambrano, A., Fontanesi, F., Solans, A., de Oliveira, R.L., Fox, T.D., Tzagoloff, A. and Barrientos, A. (2007) Aberrant translation of cytochrome *c* oxidase subunit 1 mRNA species in the absence of Mss51p in the yeast *Saccharomyces cerevisiae*. *Mol. Biol. Cell*, 18, 523-535.
3. Barros, M.H., Myers, A.M., Van Driesche, S. and Tzagoloff, A. (2005) *COX24* codes for a mitochondrial protein required for processing of the *COX1* transcript. *J. Biol. Chem.*, 281, 3743-3751.
4. Shingu-Vazquez, M., Camacho-Villasana, Y., Sandoval-Romero, L., Butler, C.A., Fox, T.D. and Perez-Martinez, X. (2010) The carboxyl-terminal end of Cox1 is required for feedback-assembly regulation of Cox1 synthesis in *Saccharomyces cerevisiae* mitochondria. *J. Biol. Chem.*, 285, 34382-34389.
5. De Silva, D., Poliquin, S., Zeng, R., Zamudio-Ochoa, A., Marrero, N., Perez-Martinez, X., Fontanesi, F. and Barrientos, A. (2017) The DEAD-box helicase Mss116 plays distinct roles in mitochondrial ribogenesis and mRNA-specific translation. *Nucleic Acids Res.*, 45, 6628-6643.
6. Barrientos, A., Zambrano, A. and Tzagoloff, A. (2004) Mss51p and Cox14p jointly regulate mitochondrial Cox1p expression in Saccharomyces cerevisiae. *EMBO J*, 23, 3472-3482.
7. Barrientos, A., Korr, D. and Tzagoloff, A. (2002) Shy1p is necessary for full expression of mitochondrial COX1 in the yeast model of Leigh's syndrome. *EMBO J*, 21, 43-52.

8. Tzagoloff, A., Capitanio, N., Nobrega, M.P. and Gatti, D. (1990) Cytochrome oxidase assembly in yeast requires the product of *COX11*, a homolog of the P. denitrificans protein encoded by ORF3. *EMBO J.*, 9, 2759-2764.
9. Perez-Martinez, X., Broadley, S.A. and Fox, T.D. (2003) Mss51p promotes mitochondrial Cox1p synthesis and interacts with newly synthesized Cox1p. *EMBO J.*, 22, 5951-5961.
10. Bonnefoy, N. and Fox, T.D. (2000) In vivo analysis of mutated initiation codons in the mitochondrial *COX2* gene of *Saccharomyces cerevisiae* fused to the reporter gene ARG8m reveals lack of downstream reinitiation. *Mol. Gen. Genet.*, 262, 1036-1046.
11. Steele, D.F., Butler, C.A. and Fox, T.D. (1996) Expression of a recoded nuclear gene inserted into yeast mitochondrial DNA is limited by mRNA-specific translational activation. *Proc. Natl. Acad. Sci. U. S. A.*, 93, 5253-5257.
12. Gruschke, S., Kehrein, K., Rompler, K., Grone, K., Israel, L., Imhof, A., Herrmann, J.M. and Ott, M. (2011) Cbp3-Cbp6 interacts with the yeast mitochondrial ribosomal tunnel exit and promotes cytochrome *b* synthesis and assembly. *J. Cell Biol.*, 193, 1101-1114.
13. Barrientos, A., Zambrano, A. and Tzagoloff, A. (2004) Mss51p and Cox14p jointly regulate mitochondrial Cox1p expression in *Saccharomyces cerevisiae*. *EMBO J.*, 23, 3472-3482.
14. Fontanesi, F., Clemente, P. and Barrientos, A. (2011) Cox25 teams up with Mss51, Ssc1, and Cox14 to regulate mitochondrial cytochrome *c* oxidase subunit 1 expression and assembly in *Saccharomyces cerevisiae*. *J. Biol. Chem.*, 286, 555-566.
15. Zeng, R., Smith, E. and Barrientos, A. (2018) Yeast mitoribosome large subunit assembly proceeds by hierarchical incorporation of protein clusters and modules on the inner membrane. *Cell Metab.*, 27, 645-656.
16. De Silva, D., Fontanesi, F. and Barrientos, A. (2013) The DEAD box protein Mrh4 functions in the assembly of the mitochondrial large ribosomal subunit. *Cell Metab.*, 18, 712-725. doi: 710.1016/j.cmet.2013.1010.1007.
17. Herrmann, J.M., Stuart, R.A., Craig, E.A. and Neupert, W. (1994) Mitochondrial heat shock protein 70, a molecular chaperone for proteins encoded by mitochondrial DNA. *J. Cell Biol.*, 127, 893-902.
18. Fontanesi, F., Soto, I.C., Horn, D. and Barrientos, A. (2010) Mss51 and Ssc1 facilitate translational regulation of cytochrome c oxidase biogenesis. *Mol Cell Biol*, 30, 245-259.
19. Ausubel, F.M., Brent, R., Kingston, R.E., Moore, D.D., Seidman, J.G., Smith, J.A. and Struhl, K. (1994), *Current Protocols in Molecular Biology*, Wiley, New York, Vol. 2, pp. 13.
20. Bustin, S.A., Benes, V., Garson, J.A., Hellemans, J., Huggett, J., Kubista, M., Mueller, R., Nolan, T., Pfaffl, M.W., Shipley, G.L. *et al.* (2009) The MIQE guidelines: minimum information for publication of quantitative real-time PCR experiments. *Clin. Chem.*, 55, 611-622.
21. Lefever, S., Hellemans, J., Pattyn, F., Przybylski, D.R., Taylor, C., Geurts, R., Untergasser, A. and Vandesompele, J. (2009) RDML: structured language and reporting guidelines for real-time quantitative PCR data. *Nucleic Acids Res.*, 37, 2065-2069.
22. Pfaffl, M.W. (2001) A new mathematical model for relative quantification in real-time RT-PCR. *Nucleic Acids Res.*, 29, e45.
23. Antonicka, H. and Shoubridge, E.A. (2015) Mitochondrial RNA granules are centers for posttranscriptional RNA processing and ribosome biogenesis. *Cell Rep.*, 10, 920-932.
24. Laemmli, U.K. (1970) Cleavage of structural proteins during the assembly of the head of bacteriophage T4. *Nature*, 227, 680-685.
25. Sambrook, J., Fritsch, E.F. and Maniatis, T. (1989) *Molecular Cloning: A laboratory manual*. Cold Spring Harbor, New York: Cold Spring Harbor Laboratory Press., NY.
26. Schiestl, R.H. and Gietz, R.D. (1989) High efficiency transformation of intact yeast cells using single stranded nucleic acids as a carrier. *Curr. Genet.*, 16, 339-346.
27. Lowry, O.H., Rosebrough, N.J., Farr, A.L. and Randall, R.J. (1951) Protein measurement with the Folin phenol reagent. *J. Biol. Chem.*, 193, 265-275.
